# Modeling the isotopic evolution of snowpack and snowmelt: Testing a spatially distributed parsimonious approach

**DOI:** 10.1002/2017WR020650

**Published:** 2017-07-20

**Authors:** Pertti Ala‐aho, Doerthe Tetzlaff, James P. McNamara, Hjalmar Laudon, Patrick Kormos, Chris Soulsby

**Affiliations:** ^1^ Northern Rivers Institute, School of Geosciences University of Aberdeen Aberdeen UK; ^2^ Department of Geosciences Boise State University Boise Idaho USA; ^3^ Department of Forest, Ecology and Management Swedish University of Agricultural Sciences Umeå Sweden; ^4^ United States Department of Agriculture Agricultural Research Service Boise Idaho USA

**Keywords:** snowmelt runoff, stable water isotopes, isotope fractionation, spatially distributed, parsimonious modeling, tracer‐aided modeling

## Abstract

Use of stable water isotopes has become increasingly popular in quantifying water flow paths and travel times in hydrological systems using tracer‐aided modeling. In snow‐influenced catchments, snowmelt produces a traceable isotopic signal, which differs from original snowfall isotopic composition because of isotopic fractionation in the snowpack. These fractionation processes in snow are relatively well understood, but representing their spatiotemporal variability in tracer‐aided studies remains a challenge. We present a novel, parsimonious modeling method to account for the snowpack isotope fractionation and estimate isotope ratios in snowmelt water in a fully spatially distributed manner. Our model introduces two calibration parameters that alone account for the isotopic fractionation caused by sublimation from interception and ground snow storage, and snowmelt fractionation progressively enriching the snowmelt runoff. The isotope routines are linked to a generic process‐based snow interception‐accumulation‐melt model facilitating simulation of spatially distributed snowmelt runoff. We use a synthetic modeling experiment to demonstrate the functionality of the model algorithms in different landscape locations and under different canopy characteristics. We also provide a proof‐of‐concept model test and successfully reproduce isotopic ratios in snowmelt runoff sampled with snowmelt lysimeters in two long‐term experimental catchment with contrasting winter conditions. To our knowledge, the method is the first such tool to allow estimation of the spatially distributed nature of isotopic fractionation in snowpacks and the resulting isotope ratios in snowmelt runoff. The method can thus provide a useful tool for tracer‐aided modeling to better understand the integrated nature of flow, mixing, and transport processes in snow‐influenced catchments.

## Key Points

- We present a novel modeling method for snowpack isotope fractionation to estimate spatiotemporal variability of isotope ratios in snowmelt
- Our parsimonious model uses two calibration parameters that alone account for the isotopic fractionation caused by snow sublimation and melt
- We provide a modeling experiment to demonstrate the model functionality and a proof‐of‐concept comparison using snowmelt isotope data

## 1 Introduction

Stable water isotopes are useful and increasingly utilized tools for inferring water flow paths and travel times in hydrological systems [*Kirchner*, 2006; *Birkel and Soulsby*, 2015]. In cold climates, the snowpack is an important intermittent storage of water [*Barnett et al*., 2005], also retaining solutes and tracers, including stable water isotopes. The isotopic composition of precipitation in the solid phase is typically depleted in heavy water isotopes (^18^O and ^2^H) compared to the mean annual signature due to colder temperatures during vapor condensation processes [*Moser and Stichler*, 1974], which leads to a situation where the accumulated snowpack and eventual snowmelt runoff is also isotopically depleted with respect to the rest of the hydrological system. The depleted isotopic chemistry of the snowpack offset creates a traceable hydrological signal at freshet, which has been used to estimate contribution of snowmelt in groundwater recharge [*Earman et al*., 2006; *Jasechko et al*., 2017], understand runoff generation processes [*Carey and Quinton*, 2004; *Laudon et al*., 2004] and to perform hydrograph separations to distinguish between old and new water in streams [*Sklash and Farvolden*, 1979; *Rodhe*, 1981; *Laudon et al*., 2002]. Though valuable work has been done in understanding snowpack isotope dynamics in snow‐influenced northern catchments, the northern region is still underrepresented in the hydrological literature [*Tetzlaff et al*., 2015]. Moreover, being able to predict the evolution of snowpack and snowmelt isotope dynamics is a prerequisite to using tracer‐aided modeling approaches in affected catchments [*Lyon et al*., 2010; *Peralta‐Tapia et al*., 2016]. Such models are increasingly used for assessing mixing processes, storage dynamics, and travel times in a wide range of catchments [*Birkel and Soulsby*, 2015] as analysis of isotopic tracers is rapidly becoming more accessible [*Berman et al*., 2009].

During winter, ice‐water‐vapor interactions can change the isotopic composition of the bulk snowpack [*Kendall and McDonnell*, 1998; *Earman et al*., 2006]. Fractionation in phase changes of sublimation/condensation and freeze/thaw during percolation through snow [*O'Neil*, 1968] has the potential to change the isotopic signal in the original snow precipitation. Isotopic variability observed in snow cores and pits is usually marked both vertically in the profile, due to persistence of isotopically different snowfall [*Moser and Stichler*, 1974; *Unnikrishna et al*., 2002; *Evans et al*., 2016], and spatially between profiles in different locations of the landscape [*Dahlke and Lyon*, 2013; *Schmieder et al*., 2016]. As a consequence, the isotopic composition of snowmelt runoff varies considerably both in space and in time [*Shanley et al*., 1995; *Carey and Quinton*, 2004; *Laudon et al*., 2004; *Dietermann and Weiler*, 2013]. Despite the high natural variability resulting from the simultaneous interaction of flow paths and isotopic fractionations in the snowpack, there are more general factors governed by the landscape characteristics and the physics of fractionation leading to systematic variability in the meltwater isotopic signature [*Gustafson et al*., 2010; *Schmieder et al*., 2016].

Sublimation from the snow surface and deeper in the snowpack can enrich the heavy isotopes in snow, because the light isotopes are preferentially removed from the liquid/solid state [*Gat and Gonfiantini*, 1981]. Over the course of winter, this can result in a shift of the bulk isotopic composition of snow [*Moser and Stichler*, 1974; *Stichler et al*., 1981; *Earman et al*., 2006]. In addition to ground snow sublimation, interception by the vegetation canopy can be an important temporary storage of snow [*Hedstrom and Pomeroy*, 1998; *Lundberg and Koivusalo*, 2003]. In northern boreal environments, coniferous evergreen trees, such as pine and spruce species, are typically responsible for the majority of snow interception [*Varhola et al*., 2010]. Sublimation of intercepted snow can significantly reduce the total amount of snow in the landscape [*Lundberg and Halldin*, 2001; *Varhola et al*., 2010] but can also act as an additional factor for isotopic fractionation enriching the isotopic composition of the residual snow eventually entering the catchment after unloading [*Koeniger et al*., 2008; *Dahlke and Lyon*, 2013]. For example, *Claassen and Downey* [1995] observed a throughfall enrichment of 2.1‰ in δ^18^O in relation to snowfall at a high altitude catchment in the Rocky Mountains, Colorado.

In addition to snow sublimation, freeze/thaw processes change the isotopic composition of snowpacks. During freeze/thaw, the bulk isotopic content of the snowpack does not change, but water with different isotopic signatures is redistributed and thereby, the isotopic stratigraphy of snow layers is somewhat altered from the original snowfall [*Taylor et al*., 2001]. Importantly, liquid water in the snowpack tends to fractionate toward isotopically lighter water in equilibrium fractionation [*O'Neil*, 1968]. Therefore, it can be expected that when water leaves the pack as snowmelt runoff, the early meltwater is isotopically depleted with respect to the remaining snowpack. Consequently, this “melt‐out” process has been observed in field [*Shanley et al*., 1995; *Soulsby et al*., 2000; *Laudon et al*., 2002; *Earman et al*., 2006], laboratory [*Taylor et al*., 2001], and modeling [*Feng et al*., 2002] studies. These studies show that the onset of snowmelt tends to be more depleted in heavy isotopes than the bulk average of snowpack, and the isotopic composition of snowpack and meltwater enriches over the cumulative period of snowmelt. The ubiquitous nature of this effect was demonstrated in *Taylor et al*. [2002]. They found typical increase of 3.5–5.6‰ from the onset to termination of snowmelt in different hydroclimatic environments across the continental U.S. They also showed conceptually how the gradual enrichment of snowmelt runoff biases isotope‐based hydrograph separation. Thus, while pioneering work in hydrograph separation used average snowpack isotopic composition as the “new water” end member [*Sklash and Farvolden*, 1979; *Rodhe*, 1981], later work has highlighted the importance of repeated sampling of snowmelt water in many locations of a catchment to improve the characterization of the new water input signal [*Laudon et al*., 2002; *Schmieder et al*., 2016]. However, such sampling is logistically challenging and in most cases the problem of not having a full picture over space and time from discrete samples remains.

Previous work [*Claassen and Downey*, 1995; *Taylor et al*., 2001; *Feng et al*., 2002] has successfully simulated the isotopic fractionation processes for both snow sublimation and melt‐out of early snowmelt in physically based one‐dimensional modeling. However, these models are highly parametrized and require detailed input data. Moreover, the models operate at the point/snow core scale, whereas the catchment‐scale modeling and hydrograph separation techniques typically require temporally resolved and, increasingly, spatially specific information on the isotopic composition of snowmelt runoff [*Fekete et al*., 2006; *Smith et al*., 2016]. To our knowledge, there are presently no modeling approaches able to produce estimates of the isotopic composition in snowmelt runoff continuous over *both space and time* that would account for the isotopic fractionation processes in snowpacks, even though the need for such methods is evident for hydrograph separation or tracer‐aided modeling studies in snow‐influenced regions [*Stadnyk et al*., 2013; *Dahlke and Lyon*, 2013; *Dietermann and Weiler*, 2013; *Schmieder et al*., 2016].

The motivation of this study was to produce a spatiotemporally consistent estimate of the isotope composition of snowmelt, which can subsequently serve as input for tracer‐aided studies in snow‐influenced environments. For this purpose, we developed spatially and temporally distributed, but still parsimonious, model to simulate (1) the isotopic enrichment of snowpack due to sublimation and (2) time‐dependent depletion of snowmelt isotopic signal. Our snow isotope simulations are coupled with a spatially distributed process‐based snowmelt and accumulation model providing, for the first time, a tool to estimate quantitatively the fully spatially distributed isotopic signals of snowmelt water at the catchment scale. Our isotope modeling approach is truly parsimonious having only two parameters to account for isotope fractionation caused by sublimation and snowmelt.

The paper is structured as follows: in section 2, we describe the model development and present its core equations. Section 3 introduces the field sites and the data we use to demonstrate the functionality of the model in two ways: in section 4, we give a synthetic simulation as an example designed to illustrate how different snow and isotope interactions are conceptualized in the model, and in section 5, we provide an empirically based proof‐of‐concept, testing the model against snowmelt lysimeter data from two long‐term experimental catchments with dominant snow influence. In section 6, we discuss the limitations of the proposed modeling approach and in conclusion section 7, we end with perspectives on how the developed model can be used to better understand the integrated nature of flow, mixing, and transport processes in snow‐influenced catchments.

## 2 Model Development

Our new simulation approach combines a process‐based snow accumulation/melt model with parsimonious calculation routines to simulate the isotopic evolution of the snowpack and snowmelt runoff. The model calculations are fully spatially distributed in grid cells using the PCRASTER PYTHON framework [*Karssenberg et al*., 2010]. The framework facilitates explicit formulation of the spatial variability in environmental forcing variables, such as radiation loading and air temperature, and landscape characteristics, such as canopy cover. As spatially explicit information, the model requires a digital elevation model (DEM) and information about the canopy structure, parameterized as canopy leaf area index (LAI). Time‐variable inputs required to drive the model are air temperature, wind speed, relative humidity and incoming shortwave radiation, precipitation, and the isotopic composition of the precipitation. Measured isotopic composition of snowpack or snowmelt runoff is beneficial for model calibration. Both the spatial (model cell size) and temporal (time step length) resolution of the model can be varied; in the work presented here, we use 100 × 100 m and 25 × 25 m grid cells and a daily time step.

Calculation of the spatially distributed process‐based snow module relies primarily on the methodology provided by *Wigmosta et al*. [1994] and *Walter et al*. [2005], building on prior work for energy balance snow modeling [*Anderson*, 1968]. The governing equation for snow accumulation and melt is

(1) $$\lambda_{f}\rho_{w}\Delta SWE=R_{net}+L_{net}+Q_{S}+Q_{L}+Q_{P}-SWE(C_{i}\Delta T_{sn})$$

where λ_*f*_ (MJ kg^−1^) is the latent heat of fusion; ρ_*w*_ (kg m^−3^), density of water; *ΔSWE* (m), change in snow water equivalent; *R*_*net*_ (MJ m^−2^), net shortwave radiation; *L*_*net*_ (MJ m^−2^), the net longwave radiation; *Q*_*s*_ (MJ m^−2^), sensible heat exchange; *Q*_*L*_ (MJ m^−2^), latent heat of vaporization or condensation at the snow surface; *Q*_*P*_ (MJ m^−2^), advective heat from rainfall; *C*_*i*_ (MJ kg^−1^ °C^−1^), specific heat capacity of ice; Δ*T*_*sn*_ (°C) is the change in snow temperature. The short and longwave radiation terms are adjusted for canopy sheltering and hillshading according to *Wigmosta et al*. [1994] and *ESRI* [2011] (see equations (S8) and (S12), part of Table S1 in supporting information S1).

The methodology to account for canopy snow interception and unloading is adopted from *Hedstrom and Pomeroy* [1998], *Pomery* [2002], and *Liston and Elder* [2006]. The calculation of the energy balance components in equation (1) and the canopy snow interception are fully described in supporting information S1. Herein, we provide a detailed description of the simulation of the isotopic composition of snowpack and snowmelt. There are three major assumptions on which the snow isotope calculation method relies on. Discussion of the implications of the simplifying assumptions in equations (2), (3), (4) is provided in section 6.

*Isotopic composition of the snowpack is fully mixed within each time step*. This simplification is based on the homogenization of the snowpack isotopes during overall melt [*Taylor et al*., 2001; *Koeniger et al*., 2008], when most of the snowmelt runoff is produced. The isotopic ratio of the ground snowpack *i*_*sn*_ (‰) is solved using the mass balance equation:

(2) $$i_{{sn}_{j}}=\frac{i_{sn(j-1)}*{SWE}_{\left(j-1\right)}+{{i_{P_{j}}*S}_{{thru}_{j}}+i}_{P_{j}}*P_{{liq}_{j}}+i_{{int}_{j}}*S_{{unl}_{j}}-i_{{snowE}_{j}}*E_{{snow}_{j}}-i_{{melt}_{j}}*S_{{melt}_{j}}}{{SWE}_{\left(j-1\right)}+\;S_{{thru}_{j}}+P_{{liq}_{j}}+S_{{unl}_{j}}-E_{{snow}_{j}}-S_{{melt}_{j}}}$$

where *j* is the simulation time step; *SWE* (mm), snow water equivalent in the snowpack; *i*_*P*_ (‰), isotope ratio in the precipitation; *S*_*thru*_ (mm), throughfall bypassing interception storage (equation (S33)); *P*_*liq*_ (mm), liquid precipitation (equation (S6)); *i*_*int*_ (‰), isotope ratio of snow interception storage; *S*_*unl*_ (mm), water unloaded from interception storage (equation (S34)); *i*_*snowE*_ (‰), isotope ratio of sublimated water from equation (3); *E*_*snow*_ (mm), amount of simulated snow sublimation (equation (S29)); *i*_*melt*_ (‰), isotope ratio of snowmelt from equation (4); and *S*_*melt*_ (mm) is the amount of snowmelt (equation (S28)). Isotope ratios are presented using the δ‐notation in reference to Vienna Standard Mean Ocean Water standards.

*Snow sublimation isotopically enriches the snowpack*. This is achieved by introducing an offset parameter to determine the level of depletion of the sublimated water relative to the snowpack:

(3) $$i_{snowE}=i_{sn}-E_{frac}$$

where *i*_*snowE*_ (‰) is the isotope ratio of the sublimated water from snow; *i*_*sn*_ (‰), isotopic concentration of the snowpack; and *E*_*frac*_ (‰) is the offset parameter. *E*_*frac*_ is a calibration parameter and is allowed to take values between 0 and 15‰ based on the equilibrium difference of 15‰ between ice and vapor isotopic ratio at 0°C temperatures [*Ellehoj et al*., 2013]. The formulation results in isotopic exchange between the atmosphere and the snowpack whenever sublimation or condensation is simulated (equation (S29)), and the magnitude of exchange depends on the value of *E*_*frac*_ parameter.

The onset of overall snowmelt is isotopically depleted with respect to the average snowpack, and the difference reduces over time. This is achieved by using an offset parameter *M*_*frac*_ assigning a more depleted value for the initial snowmelt, and tracking the number of days with simulated snowmelt *d*_*melt*_ runoff to reduce the offset over time:

(4) $$i_{melt}=i_{sn}-\frac{M_{frac}}{d_{melt}}$$

where *i*_*melt*_ (‰) is the isotope ratio of the snowmelt water; *M*_*frac*_ (‰), offset parameter; and *d*_*melt*_ is the number of days snowpack has experienced snowmelt. *M*_*frac*_ is a calibration parameter taking values between 0 and 3.5‰ based on the equilibrium difference of 3.5‰ between ice and liquid water isotopic ratio [*Gat and Gonfiantini*, 1981]. The following constraints apply when the snowmelt depletion is simulated, i.e., the parameter *d*_*melt*_ ≥ 1, are used: (i) SWE > 10 mm, with the analogy that the snowpack needs to be sufficiently deep to allow contact time between ice and water leading to isotopic fractionation, (ii) the snowmelt runoff flux need to exceed a threshold of 2 mm d^−1^, with the aim to simulate the evolution of snowmelt primarily during the overall melt and to ignore minor melt events in the beginning and middle of the snow covered period. If these conditions are met, the parameter *d*_*melt*_ is cumulatively increased by one for each melt day, leading to a progressively lower difference between isotopic composition of snowmelt and the remaining snowpack.

The isotopic composition of interception storage *i*_*int*_ (‰) is solved from

(5) $$i_{{int}_{j}}=\frac{i_{int(j-1)}*I_{sn(j-1)}+i_{P_{j}}*P_{{ice}_{j}}-i_{{intE}_{j}}*E_{{int}_{j}}}{I_{sn(j-1)}+P_{{ice}_{j}}-E_{{int}_{j}}}$$

where *I*_*sn*_ (m) is the interception storage and *i_intE_* (m) is the isotopic composition of water sublimated from the interception storage with the sublimation fractionation offset parameter *E*_*frac*_ included as in equation (3). Sublimation from interception storage *E*_*int*_ is approximated from latent heat exchange with the same equations as for ground snowpack (equations (S20)–(S23) and (S29)), except the snow temperature (*T*_*s*_) in the interception storage is assumed to equal air temperature *T_a_* and the measured wind speed W is used instead of wind speed *W*_*can*_ reduced by canopy effects (equation (S3)).

## 3 Study Sites

**Figure 1:**
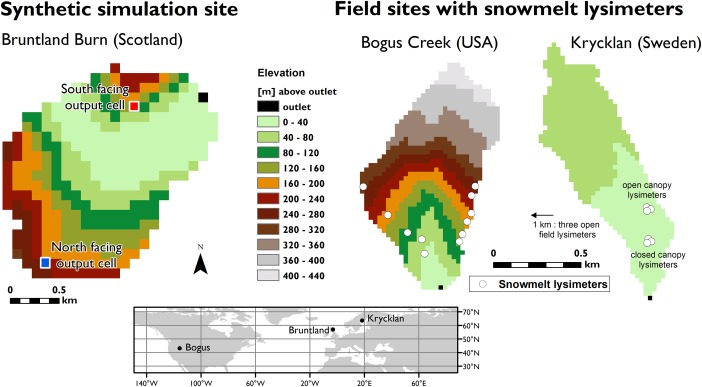
(left) The Bruntland Burn catchment (3.2 km^2^) was used for the synthetic modeling experiment. The model cells on south and north facing slopes serve as locations where model output is extracted to compare the influence of landscape orientation (i.e., difference in radiation exposure and temperature). (right) Snowmelt lysimeter data from Krycklan and Bogus Creek (0.5 and 0.6 km^2^, respectively) were used as a “proof‐of‐concept” test for the model. In all figures the color scheme for elevation is the same to illustrate differences between catchments, and black cell shows the catchment outlet.

To demonstrate functionality of the model outlined above, we used data from three long‐term experimental catchments (Figure 1): Bruntland Burn in the Scottish Highlands, Krycklan C7 in Swedish boreal forest and Bogus Creek, a headwater subcatchment of the Dry Creek experimental watershed in Idaho, USA. The catchments were utilized in two ways.

The Bruntland Burn catchment was only used as a platform for a synthetic modeling experiment to demonstrate how the model functions in simulating spatiotemporally distributed snow sublimation and melt as well as the isotopic transformations involved. The modeling experiment was driven by averaged climate data from the Krycklan catchment. The modeling experiment setup along with its results and discussion are presented in section 4.

Krycklan and Bogus catchments were further used to test the model output against field data of snowmelt runoff collected with snowmelt lysimeters. The details for the model calibration at the sites with the modeling results and discussion are presented in section 5.

Here we briefly describe the relevant topographic, climatic, and canopy characteristics that influence snow accumulation and melt at the catchments used in empirically based model testing (Krycklan and Bogus). We also explain the climatic data used to drive the simulations and snowmelt lysimeter data used in model calibration and testing. For a more comprehensive description of the catchments, the reader is referred to the work cited below.

### 3.1 Krycklan Catchment Characteristics and Model Data

The Krycklan catchment (0.5 km^2^), located in the Swedish boreal forest (Figure 1), is a well‐established experimental site for hydrology and biogeochemistry research [*Laudon et al*., 2013]. It has a gentle relief with altitudes ranging from 235 to 306 m. Annual average precipitation (P) is 622 mm, approximately 35–50% of which falls as snow [*Laudon and Löfvenius*, 2016]. Annual average air temperature (T) is 2.4°C with subzero monthly mean temperatures and snow cover typically during November–March. Snowmelt occurs between April and May. Most of the land cover (82%) is conifer boreal forest (*Pinus sylvestris* and *Picea abies*) with a part of the catchment covered by a canopy‐free minerogenic mire (18%) dominated by Sphagnum moss.

**Figure 2:**
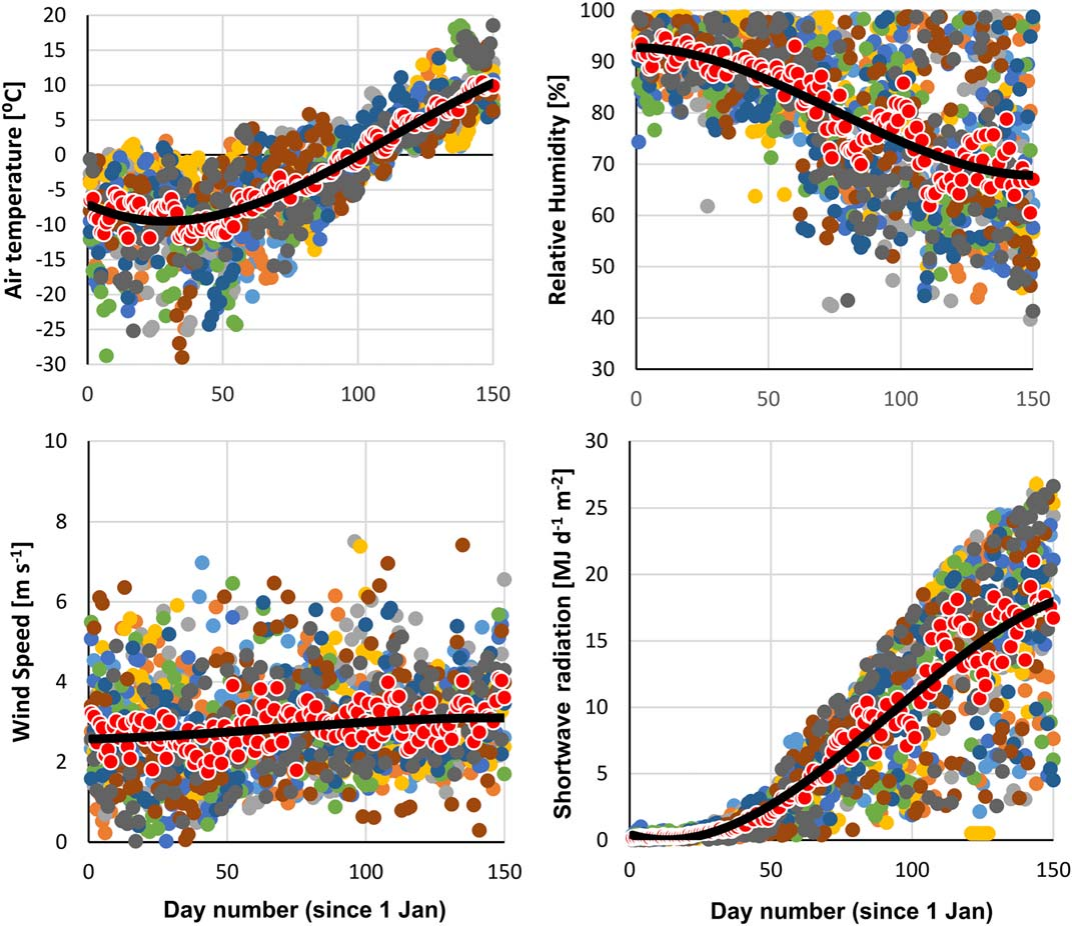
Climate variables from the Krycklan site, used as input for the synthetic simulation exercise. Colored circles are climate observation for individual days over 11 years (2002–2013), red circles are the averages for a given day of the year and the solid black line is the smoothed average which serves as the synthetic simulation input data for each climate variable.

For model inputs, a spatially distributed LAI of 2 was assigned for conifer forest stands, approximating a typical LAI value for conifer canopies present at the site [*Rasmus et al*., 2016], and a value of 0 was given for the canopy‐free mire. For climate data (daily P, T, shortwave radiation, relative humidity, and wind speed), we used the Svartberget meteorological station adjacent to the catchment. Long‐term averages of these data were used in the synthetic simulation example (Figure 2), and here we utilize the same time series to perform simulations from 1 January 2002 to 31 December 2012. Precipitation (both rain and snow) has been sampled for isotopes on an event basis in Krycklan since 2002, providing a uniquely long and consistent data set for precipitation isotopes for model input [*Peralta‐Tapia et al*., 2016]. Neither climate nor isotope data series were corrected for elevation effects, because they are negligible given the small elevation range [*Karlsen et al*., 2016].

Snow water equivalent (SWE) in Krycklan was measured starting every midwinter and repeated at approximately 2–3 week intervals until the snow had melted [*Laudon and Löfvenius*, 2016]. Measurements were carried out with a snow corer tube in three adjacent locations with three replicates each, in an open area 1 km west from the study catchment. The isotopic composition of snowmelt was sampled with nine 1.44 m^2^ snowmelt lysimeters, with three replicates in each dominant land cover types: pine forests (open canopy), spruce forest (closed canopy), and open area (Table S2 in supporting information S1). Location of open and closed canopy lysimeters is given in Figure 1, the open area lysimeters were situated at the SWE measurement site. Sampling occurred over three springs: 99 samples during 14 occasions in 2004, 74 samples during 12 occasions in 2010, and 86 samples during 12 occasions in 2012. Mean and standard deviation for each sampling are shown in Figure 4, full details of the sampling and the analytical method can be found in *Peralta‐Tapia et al*. [2016].

### 3.2 Bogus Catchment Characteristics and Model Data

The Bogus catchment is a small headwater (0.6 km^2^) in the Dry Creek experimental watershed (DCEW) where a V‐shaped fluvial valley slopes steeply from 1684 to 2135 m asl. Several field and simulation studies in the wider DCEW have focused on understanding the spatially varying snow distribution and melt processes [*Kelleners et al*., 2010; *Homan et al*., 2011; *Eiriksson et al*., 2013; *Kormos et al*., 2014; *Evans et al*., 2016]. The site receives about 670 mm of P annually, with more than 50% occurring during winter as snowfall, the percentage increasing with altitude. Average annual T is 8.8°C with below‐zero mean monthly temperatures from November to March in the highest parts of the catchment, and December to February at the catchment outlet. Shrubs (*Prunus* spp. and *Ceanothus* spp.) cover the most of the catchment, with a small fraction of taller tree canopies (*Pseudotsuga menziesii* and *Pinus ponderosa*) focused primarily in the valley bottom near the stream.

LAI was parameterized using a spatially distributed map of vegetation height from a LIDAR survey. A LAI of 0 was assigned to vegetation height < 1 m to exclude interception processes for the low shrub vegetation. LAIs of 2 and 1.5 were assigned for canopies over 3 m and between 1 and 3 m, respectively. We used climate data from the Treeline meteorological station located 4 km south‐east and roughly the same altitude as the catchment outlet. Occasional gaps were filled with data from the SNOTEL meteorological station no. 978 [*National Climatic Data Center*, 2016] located 200 m north of the catchment. Because of the prominent elevation gradient, a spatially distributed environmental lapse rate of −0.6°C 100 m^−1^ was applied to T according to moist adiabatic lapse rate. To P a +5.4% 100 m^−1^ increase was established from field measurements in the Bruntland Burn [*Ala‐aho et al*., 2017], and the parameter value was transferred to the Bogus site.

Event‐based isotope samples for P were not available in Bogus. To construct a continuous isotope input with daily resolution, we used samples taken in the DCEW between 2003 and 2012 (n = 142) to build a linear regression model to estimate continuous a time series for precipitation from daily T similarly as in *Tappa et al*. [2016]. In addition, we applied an environmental lapse rate of −0.22‰ for δ^18^O per 100 m rise in elevation established for the DCEW [*Tappa et al*., 2016].

SWE data for Bogus were acquired from the SNOTEL (same as for meteorological data) station where SWE is measured continuously with a pressure transducer [*National Climatic Data Center*, 2016]. Snowmelt water was sampled for isotopes in the winter 2002/2003 using twelve 19 L. melt buckets (Table S2 in Supporting Information S1). The melt buckets were installed in autumn 2002 before snow accumulation, and sampled during the winter 2003. The buckets were positioned along two transects: five buckets along a western slope covering an elevation gradient of 1718–1983 m, and seven buckets along the eastern ridge between 1807 and 1985 (Figure 1). The total data set consist of 87 samples taken on 12 occasions between 11 January and 14 April 2003. Full details for the sampling design, variability in each location, and analytical methods are given in *Kormos* [2005].

## 4 Synthetic Modeling Experiment Demonstrating Model Functionality

### 4.1 Setup of the Modeling Experiment

To demonstrate how the model functions in simulating spatiotemporally distributed snow sublimation and melt as well as the isotopic transformations involved, we devised a synthetic simulation based on a “thought experiment.” The aim of the exercise was to highlight how our simulations take into account different landscape position (aspect and altitude) and canopy cover—both shown to be potentially influential for snow accumulation and melt [*Carey and Quinton*, 2004; *Varhola et al*., 2010] and related isotopic processes [*Koeniger et al*., 2008; *Dahlke and Lyon*, 2013; *Schmieder et al*., 2016]. To showcase our model functionality, we extracted model output variables from two landscape positions with different radiation exposure due to aspect: north and south facing slopes (Figure 1), and we performed two simulation scenarios: with and without tree canopy. With the scenarios, we explored a hypothetical situation where a midwinter snowpack was subjected to climate conditions of progressively increasing air temperature, radiation loading and reduced relative humidity—typical changes as winter turns to spring in these higher latitudes.

For the modeling experiment, we used a digital elevation model of the Bruntland Burn (Figure 1): a 3.2 km^2^ catchment in the Scottish Highlands [*Soulsby et al*., 2015] with north and south facing slopes and a moderate elevation gradient from 250 to 530 m asl, which allowed us to demonstrate the effect of landscape orientation on the simulation output (Figure 1). Using this catchment as a platform, we also utilized a long‐term climatic data set from Krycklan [*Laudon et al*., 2013] (see section 3). These conditions provided climate data encompassing a period of continuous subzero temperatures and a month‐long distinct spring snowmelt, which allowed us to test the model functionality in both winter and spring. The site also had a wealth of isotope data for precipitation, snowpack and snowmelt [*Laudon et al*., 2013; *Peralta‐Tapia et al*., 2016; *Laudon and Löfvenius*, 2016]. The numerical experiment proceeded as follows:

1. We preassigned model cells on south and north facing slopes in the Bruntland (Figure 1) to serve as locations where the model output was extracted to compare the influence of landscape orientation.
2. We initialized the model with a snowpack of 200 mm and an isotopic composition of −25‰ in δ ^18^O uniformly in the catchment, both values feasible for a midwinter snowpack at the Krycklan climate [*Laudon and Löfvenius*, 2016]. Parameters *E*_*frac*_ and *M*_*frac*_ were given equilibrium fractionation values of 15‰ and 3.5‰ [*Gat and Gonfiantini*, 1981; *Ellehoj et al*., 2013], respectively, to allow for maximum fractionation effects.
3. Using 11 years of daily climate data, we calculated an average for each day of the year, and fitted a polynomial function through these averaged data to represent a “smooth evolution” of the climatic transition from winter to spring (Figure 2). These smoothed averages starting 1 January and continuing until 30 May (150 days) were used as the model input data.
4. We ran the model with the averaged climate drivers and extracted model outputs for the north and south facing slopes for four variables: water stored in the snowpack (SWE) (mm), isotopic composition of the snowpack (δ^18^O ‰), snowmelt flux exiting the snowpack (mm d^−1^), and isotopic composition of the snowmelt water (δ^18^O ‰).

To additionally demonstrate how tree canopy influences the simulations, we repeated the steps above, with the exception of adding a tree canopy with LAI of 3 and canopy cover of that can be considered to be representative of a dense mature boreal conifer forest [*Rasmus et al*., 2016], to demonstrate impacts in closed conifer canopy. We changed the model initial conditions in step 2 so that the initial snowpack of 200 mm was split between interception storage (88 mm) and ground snow storage (112 mm) with the purpose of satisfying a full interception storage according to equation (S32) to maximize the interception effects in the canopy scenario, yet with empirically feasible values [*Hedstrom and Pomeroy*, 1998].

### 4.2 Simulation Output Demonstrating the Functionality of the Coupled Snow Isotope Model

**Figure 3:**
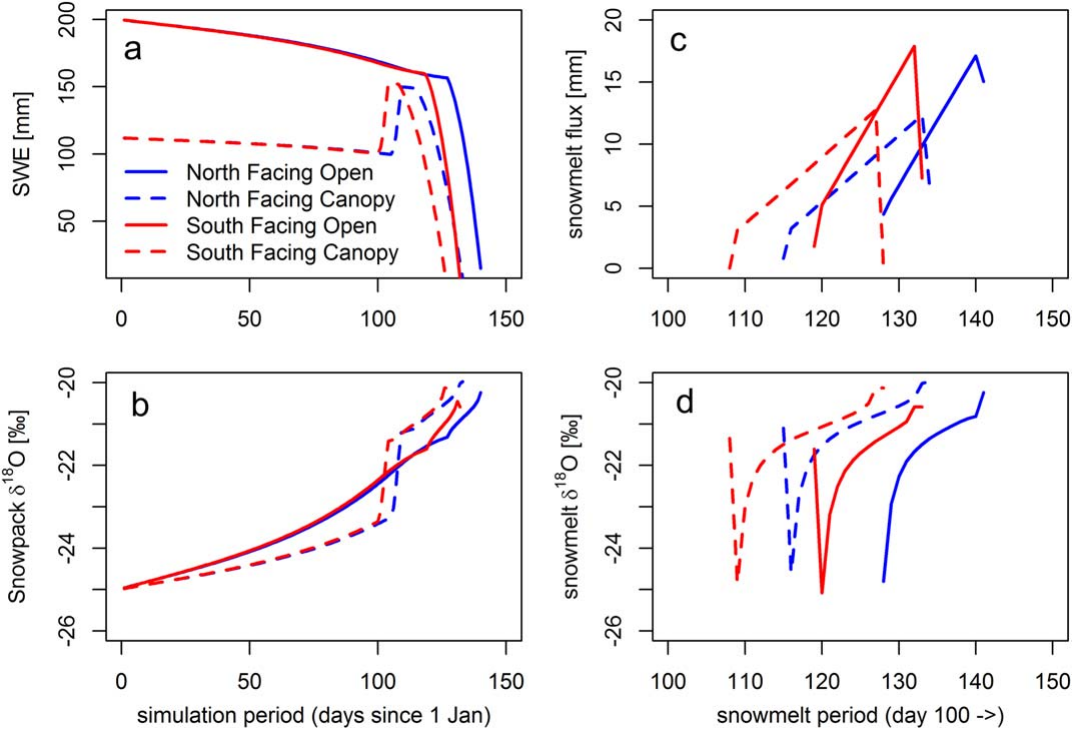
Output from the synthetic simulation example from north and south facing slopes (Figure 1), both with a canopy‐covered and open scenario. (a) The SWE in ground snowpack, (b) the isotopic composition of ground snowpack, (c) the snowmelt flux during the snowmelt period (days 100–150), and (d) the isotopic composition of the snowmelt.

Running the model with the smoothed climate input data in Figure 2 allowed us to assess how the model stores and releases water to/from the snowpack and what the isotopic composition of these storages and fluxes are. Our synthetic example (Figure 3) highlights how (i) the model simulates the ground snow and interception sublimation that have the potential to enrich the isotopic composition of snowpack and (ii) how the model enriches the isotopic composition during snowmelt (“melt‐out” phenomenon). Previous modeling work has successfully simulated snowpack isotopic fractionation processes due to both sublimation and snowmelt with more detail and physical rigor in 1‐D simulations [*Claassen and Downey*, 1995; *Taylor et al*., 2001; *Feng et al*., 2002]. Our model adds to the literature by presenting a truly parsimonious approach that allows fully spatially distributed estimates of snowmelt isotopes by incorporating only two parameters, *E*_*frac*_ and *M*_*frac*_, to an otherwise generic process‐based spatially distributed snow model.

Snow storage in the ground snow (Figure 3a) is progressively reduced for the first 100 days due to snow sublimation. Sublimation rates on south and north facing slopes in the “no canopy” scenario show similar decline rates with 32 mm of snow sublimated by day 100, giving an average rate of snow sublimation of 0.32 mm d^−1^. In the “canopy” scenario, the sublimation rate from ground snowpack is smaller (12 mm), with an average rate of 0.12 mm d^−1^. Around day 100, the interception storage is unloaded due to above zero air temperatures, according to equation (S34). After the unloading, the ground snow storage is lower than in the “no canopy” scenario, giving a higher combined sublimation rate of 0.47 mm d^−1^ from ground snow and interception storage than ground snow storage alone.

The isotopic melt‐out process from the snowpack is well documented in the literature, but there is less work on the sublimation enrichment of snowpacks. Nevertheless, the influence of sublimation on the isotopic composition of snow has been convincingly shown [*Moser and Stichler*, 1974; *Stichler et al*., 1981], and therefore should be accounted for in any calculation attempts to estimate isotopic composition of spatially distributed snowmelt runoff. This is particularly important at sites with significant differences in solar forcing [*Gustafson et al*., 2010] or significant snow interception on evergreen canopies [*Koeniger et al*., 2008]. Laboratory experiments [*Moser and Stichler*, 1974] observed snow sublimation to cause enrichment of 0.2‰ per percent weight sublimated. In our simulation example with 55 mm (27%) of snow sublimated in the “no canopy” scenario, this would equate to a theoretical enrichment of 5.4‰, which exceeds our simulated value of ∼3.5‰. More importantly, our simulation example highlights how the interception storage can add to the total isotopic fractionation of snowpack. *Koeniger et al*. [2008] found an enhanced isotopic enrichment of ∼0.2‰ per unit increase in LAI, which agrees with our additional enrichment of 0.7‰ in the scenario when LAI is increased from 0 to 3 (Figure 3).

The effect of snow sublimation on the ground snow isotopic composition (Figure 3b) shows an enrichment of 2.6‰ in the “no canopy” scenario and 1.6‰ in the “canopy” scenario by day 100. The difference of 1.0‰ is caused by the lower ground snow sublimation rates under the tree canopy. The interception storage creates an additional avenue of fractionation, which is seen as steeply elevated snowpack enrichment during snow unloading, leaving the ground snow 0.7‰ more enriched due to interception enrichment.

Air temperature exceeds 0°C on day 101 (Figure 2; at the catchment outlet, later at higher elevations due to temperature lapse rate (Figure 1)), which marks the initiation of snowmelt in the catchment. After breaking the 0°C threshold, there is an 8–28 day delay, after which the snowpack begins to release water (Figure 3c). The initial snowmelt is retained in the snowpack and the length of the delay depends primarily on the landscape location and canopy scenario: melt initiates ∼10 days earlier with canopy compared to open scenario, and ∼10 days earlier in south facing compared to north facing slopes. Melt rates are linearly increasing, with higher rates and thereby shorter snowmelt periods in the “open” scenarios. A decrease of the melt flux on the last day of melt is caused by having only the residual snow available for melt.

Earlier snow ablation in the canopy‐covered scenarios is somewhat counterintuitive, because canopy sheltering can postpone completion of snowmelt [*Marks and Winstral*, 2001; *Rinehart et al*., 2008]. In the simulated “canopy cover” scenario, the initial 200 mm SWE storage is split between a ground snowpack (112 mm) and a full interception storage (88 mm). When the air temperature rises above freezing on day 101, in comparison to the “open” scenario, the “canopy” scenario ground snowpack (1) has less snow that needs warming before it becomes isothermal at 0°C and melt initiates, (2) receives rapid unloading of snow from interception storage with 0°C temperature that warms the ground snowpack, and (3) has less snow after the unloading, because of sublimation from the interception storage. The above reasons combined, along with potential overestimation of canopy‐emitted longwave radiation due to the simplifying assumption that canopy temperature equals air temperature (equation (S13)), result in earlier ablation of snow in the “canopy” scenario of our numerical experiment.

Because in three of the simulations a threshold flux of 2 mm d^−1^ was not exceeded to start the melt‐out process (see equation (4)), the first day of melt is assigned the average snowpack composition on the day (Figure 3d). When the threshold flux is exceeded after the second day of melt, the isotopic composition of early snowmelt starts from ∼–24.5‰ in all scenarios, the minor variability caused by differences in simulated snowpack composition. From there on, the isotopic composition of meltwater gradually enriches, finishing between −4‰ and −4.5‰ more enriched compared to the initial melt. Although the simulation experiment does not aim to reproduce any specific field observations, the modeled ranges for isotopic evolution from first melt event to snowpack exhaustion (∼4–4.5‰) are in line with field studies demonstrating similar ranges of evolution during overall melt ∼4.4‰ [*Laudon et al*., 2002], ∼4‰ [*Taylor et al*., 2001], 3.5–5.6‰ [*Taylor et al*., 2002], and ∼3.5‰ [*Shanley et al*., 1995]. Furthermore, the shape of the depletion curve (Figure 3d) closely resembles the empirically derived logarithmically increasing evolution shown in controlled laboratory environments and numerical simulations [*Taylor et al*., 2001; *Feng et al*., 2002]. Under field conditions, the shape of melt‐out curve would be distorted because of day‐to‐day variations in the weather (see Figure 2), but our simulation experiment with averaged input data allows the melt to progress linearly as if in a controlled environment, and therefore more clearly demonstrates the process dynamics in the model.

## 5 Model Test Against Measured SWE and Snowmelt Lysimeter Data

### 5.1 Model Calibration

To provide a test of the model, performance was assessed against measured snowmelt lysimeter data for four winters/spring transitions. The snowmelt samples are collected at two experimental catchments with a strong snow influence, but contrasting winter conditions: (1) Bogus Creek located at snow/water transition zone in Idaho, USA and (2) Krycklan located in the Swedish boreal forest with a colder winter and a more persistent snow cover (model data are presented in section 3).

The measured SWE and snowmelt lysimeter data were used in the model calibration. In Bogus, the observed daily SWE time series were matched to the simulation output extracted from the highest and most northern model cell, which was ∼200 m south from the SNOTEL station. In Krycklan, we used the global average of the three measurement locations where SWE was determined and matched that with the model output extracted for the open mire area corresponding to the conditions of the measurement location.

To compare the simulated isotopic composition of snowmelt to that of actual snowmelt samples, we extracted the spatially distributed simulated meltwater flux and its isotopic composition for each time step. From these data, we calculated the flow‐weighted catchment average of meltwater isotopic composition *i*_*fw*_ (‰) with

(6) $$i_{fw}=\frac{\sum_{N_{cell}}^{j=1}W_{{out}_{j}}\times i_{{melt}_{j}}}{\sum_{N_{cell}}^{j=1}W_{{out}_{j}}}$$

where *W*_*out j*_ (m) is the snowmelt runoff from the snowpack in a given cell; *i*_*melt j*_ (‰), isotope ratio of the snowmelt water in a given cell; and *N*_*cell*_ is the number of model cells in the catchment.

Because snowmelt lysimeters integrate the isotopic signal of snowmelt between the sampling days, for comparison purposes we calculated another flow‐weighted sum *i*_*comp*_ to weight the simulated isotope ratio in snowmelt with the simulated flux of snowmelt water between lysimeter sampling days:

(7) $$i_{comp}=\sum_{M_{k+1}}^{M_{k}}\frac{\sum_{N_{cell}}^{j=1}W_{{out}_{j,k}}*i_{{fw}_{k}}}{\sum_{N_{cell}}^{j=1}W_{{out}_{j,k}}}$$

where *M*_*k*_ is the day number of snowmelt lysimeter measurements (in relation to simulation output time steps 1–150), and k goes from 1 to total number of lysimeter sampling days at each site.

The steps above resulted in pairs of observed and simulated values for both SWE and isotopic composition of snowmelt. We performed 10,000 Monte Carlo simulations varying six parameters in order to find parameter values which resulted in good agreement between observed‐simulated pairs. Varied parameters and their ranges were *M*_*frac*_ (0–3.5‰, in equation (4)), *E*_*frac*_ (0–15‰ in equation (3)), *c*_*corr*_ (0–0.3, correction coefficient for snowfall undercatch in addition to *Yang* [1998] in equation (S5)), *TT*_*low*_ (–2 to 0°C, threshold temperature below which all precipitation is ice equation (S1)), *TT*_*high*_ (0–2°C, threshold temperature above which all precipitation is liquid equation (S1)), and *a*_*pow*_ (0–3, parameter accounting for the decline of the albedo in old snow equation (S9)). The first two influence the level of isotopic fractionation during snowmelt and sublimation, respectively, and the latter four affect the snow accumulation and melt processes supporting information (S1).

We used a single goodness‐of‐fit (GOF) metric for both observation‐simulation pairs to differentiate between rejected model runs and those accepted as “behavioral”. The Kling‐Gupta efficiency statistic (KGE) [*Gupta et al*., 2009] was used for SWE and mean absolute error (MAE) for the snowmelt isotope ratios. From the ensemble of 10,000 model runs, the 100 “best” runs were selected using the cumulative distribution function (CDF) of the GOF measures as in *Ala‐aho et al*. [2017]. Each parameter set, and the resulting simulation output, maps a value on the CDF of the GOF measure for both calibration variables (KGE for SWE; MAE for isotope ratios). We identified a threshold quantile *q*_*thres*_ in the CDF that maps exactly 100 simulations above it for SWE where the aim to is find high KGE values, and 1 – *q*_*thres*_ for MAE, where we aim to find low error values. The 100 model runs for which the GOF was above *q*_*thres*_ (for KGE) and below 1 – *q*_*thres*_ for MAE were then retained as the behavioral model runs. We used “dotty plots” [see e.g., *Beven and Freer*, 2001] to visualize model response surfaces and highlight parameter ranges resulting in behavioral simulations.

### 5.2 Simulated Isotopes in Snowmelt Runoff Against Snowmelt Lysimeter Data

**Figure 4:**
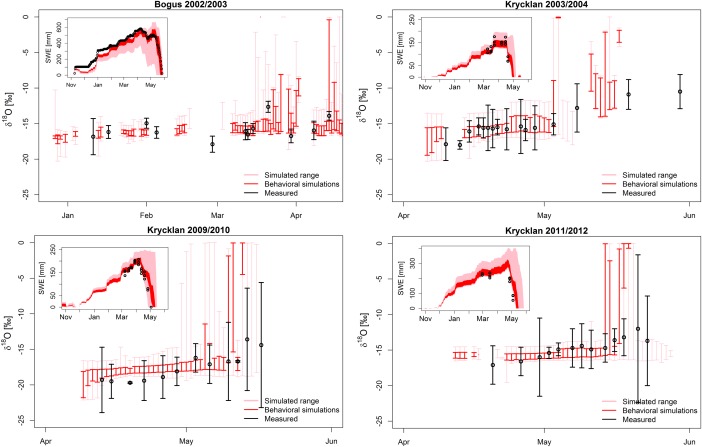
The total and behavioral range (100 best runs) of the catchment averaged isotopic compositions given in pink and red, plotted with mean and standard deviation of the snowmelt lysimeter measurements. (top left) The results in Bogus, the others show the three simulated winters in Krycklan. For each figure, the top left corner gives the measured and simulated SWE.

**Figure 5:**
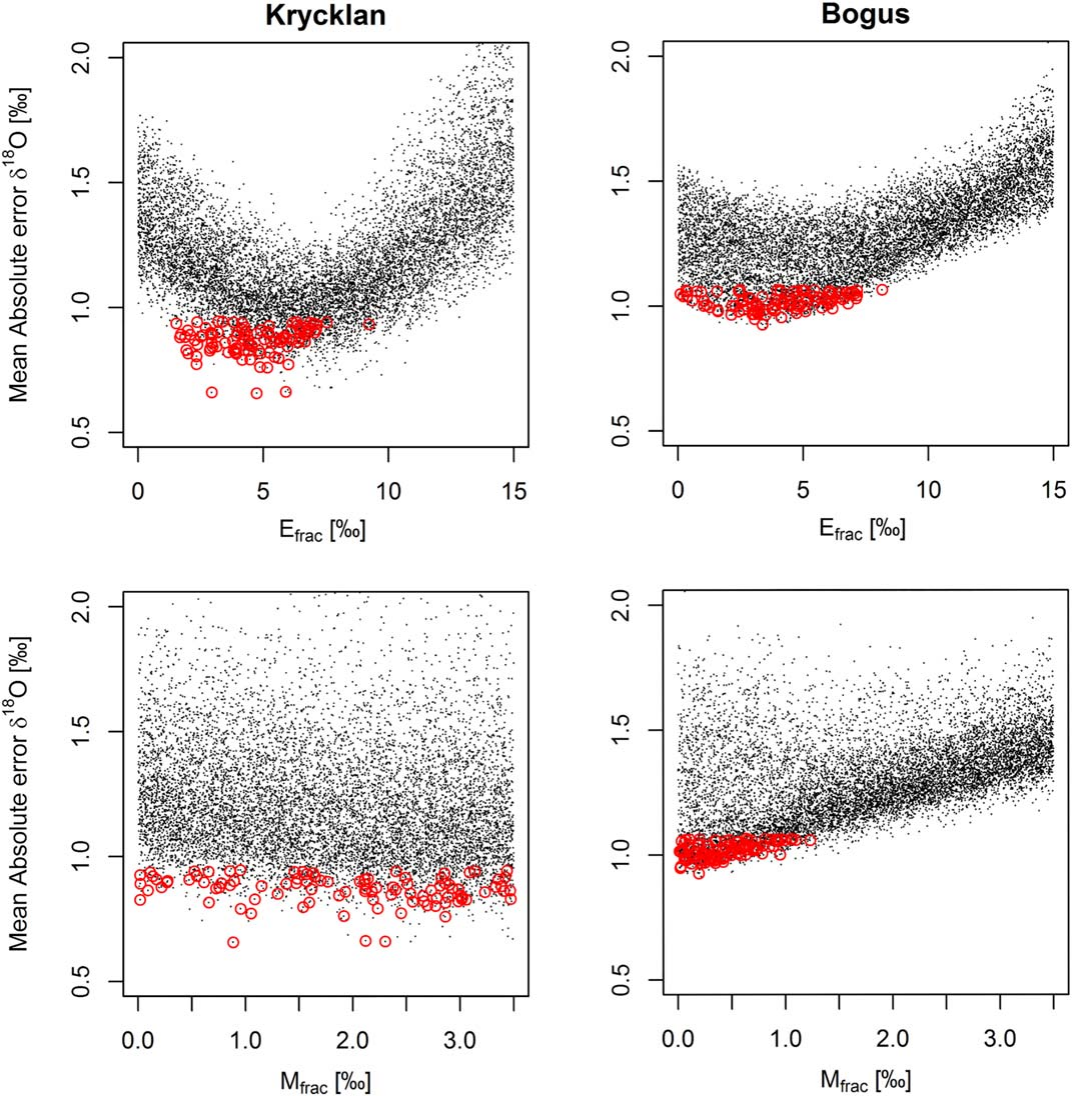
Dotty plots showing the sampled parameter ranges in all 10,000 simulations in black dots. Red circles highlight the parameters in the 100 behavioral model runs.

Our model can match the overall level of depletion, the trend of progressively enriching meltwater and to some extent the variability of the snowmelt isotopic composition for four winters at the two field sites (Figure 4). Typical absolute mean errors between simulated, catchment averaged snowmelt isotopes and observed mean values are between 0.7 and 1.0‰ in Krycklan and 0.9 and 1.1‰ in Bogus (Figures 4 and 5). A prerequisite for capturing isotopes in snowmelt is to adequately represent the snow accumulation and melt, which is successfully done by the model with KGE values for SWE between 0.6 and 0.8 in Krycklan and around 0.85 in Bogus (Figure 5).

The ranges for behavioral parameter values for the isotope fractionation parameters *E*_*frac*_ and *M*_*frac*_ are visualized for both catchments as dotty plots (Figure 5). The parameter regulating the level of sublimation enrichment of snow (*E*_*frac*_) leads to lowest errors between values −2.5 and −7.5‰ in both catchments, demonstrating the relevance of incorporating snow sublimation fractionation at both sites. In Krycklan, the parameter affecting the intensity of melt‐out process (*M*_*frac*_) produces low errors throughout the sampled range, whereas in Bogus values close to zero result in low errors, suggesting that the melt‐out process was not apparent for the simulated winter in Bogus. Parameters influencing snow accumulation and melt are not shown, but of them *a*_*pow*_ and *c*_*corr*_ were identifiable in both catchments, whereas the threshold temperatures showed lesser sensitivity.

Overall, our empirically based “proof‐of‐concept” test against field data shows how the model is capable of capturing the level and progression of the catchment averaged isotopic composition of snowmelt water in two different snow environments (Figure 1). Field studies have revealed a tremendous spatiotemporal variability in isotopic composition of snowmelt runoff. We would not expect our parsimonious modeling method to capture the full point‐scale variability present at our field sites, and therefore, instead of point‐by‐point comparison, we compared catchment averaged simulated values with averaged lysimeter measurements.

The model matches the initial composition of depleted snowmelt runoff, i.e., the simulated values prior the first lysimeter sample each year, remarkably well. This is attributed to the parameterization of snow sublimation (equation (3)), which is able to enrich the snowpack—the level of enrichment depending on the value assigned to the parameter *E*_*frac*_. The optimal range for the parameter (Figure 5) is between −2.5 and −7.5‰ with a clear U‐shape, meaning that if the sublimation process is excluded (equivalent to assigning *E*_*frac*_ a value 0) the model mean error would increase. The optimum range for *E*_*frac*_ is more consistently greater than zero and more identifiable in Krycklan than in Bogus. This may be caused by more canopy cover in Krycklan allowing additional fractionation through sublimation from the interception storage (as shown conceptually in Figure 3b). Only minor influence of sublimation fractionation at the Bogus site also somewhat agrees with the work by *Evans et al*. [2016] who did not find evidence of snow sublimation fractionation in their snow samples from the site. It should be noted that the input data for snowfall isotopes for the Bogus site were not as comprehensive as for Krycklan, which introduces more uncertainty in the model input and the subsequently in the model parameterization. All and all, the calibration results demonstrate the importance of considering snow sublimation enrichment in catchment scale shown experimentally in, e.g., *Koeniger et al*. [2008] and *Gustafson et al*. [2010], but thus far not addressed in any catchment‐scale estimates for snowmelt runoff isotopes [*Stadnyk et al*., 2013; *Delavau et al*., 2017].

The gradual enrichment of meltwater as spring progresses is evident in the lysimeter data set for Krycklan, but less so in Bogus (Figure 4). One reason for this may be that for the sampling in Bogus was conducted during midwinter snowmelt events between January and April, whereas in Krycklan, the sampling was focused during the entire melt period from April to June, with no observed midwinter melt events. Several experimental studies suggests that the isotopic composition in midwinter snowmelt runoff is less markedly affected by isotopic fractionation processes in the snowpack, because first surficial melt events and rain on an unripened snowpack may travel through the permeable snowpack as preferential “finger” flow with little interaction with the bulk snowpack [*Unnikrishna et al*., 2002; *Evans et al*., 2016; *Juras et al*., 2016]. The absence of the melt‐out process in the lysimeter data is reflected in the *M*_*frac*_ value of the behavioral simulations clustering close to value of 0 (Figure 5), showing that the model error is lowest when the melt‐out process is not simulated.

In Krycklan, the melt‐out process is more strongly evident in all years (Figure 4), as expected from prior isotope work in the catchment [*Laudon et al*., 2002, 2004]. The simulations capture the enrichment best in the spring of 2004, whereas in the springs 2010 and 2012 the simulated signatures are increasing but cannot fully match the total level of observed enrichment. Still, the behavioral simulations typically envelope the observed mean, except for April 2014. It should be pointed out that in Krycklan we calibrated the simulations to match SWE and lysimeter isotopes across all three winters, with the aim of finding universal parameter values which would be required in multiyear tracer‐aided modeling applications like done in *Ala‐aho et al*. [2017]. If the model had been calibrated to a data set for a single year as in Bogus, the model fit in each year would have improved. The trade‐off is seen in overestimation in spring 2010 and underestimation in spring 2012.

## 6 Discussion for the Limitations and Simplifying Assumptions of Isotopic Mixing and Fractionation and SWE Modeling

As evident in the equations (2), (3), (4), our modeling approach accounts for the isotopic fractionation and mixing in snowpack in a highly simplistic manner in comparison to the known process complexity established in empirical and modeling studies [*Gat and Gonfiantini*, 1981; *Claassen and Downey*, 1995; *Taylor et al*., 2001]. The most obvious contradiction with field observations is assuming complete isotopic mixing of the snowpack in each time step, whereas snowpacks are known to maintain their isotopically layered structure for most of the snow season [*Unnikrishna et al*., 2002; *Evans et al*., 2016]. However, the snowpack is considerably homogenized during ripening due to isotopic exchange of water percolating through the snowpack and diurnal melt/refreeze processes within the pack [*Taylor et al*., 2001; *Koeniger et al*., 2008; *Eskelinen et al*., 2016]. This gives a justification for our assumption, because the majority of snowmelt runoff occurs during the late season overall melt, when the snowpack is isothermal at 0°C and subject to mixing. Nonetheless, the model's capability to simulate the isotopic composition in snowmelt runoff—particularly in midwinter rain‐on‐snow events on unripened and cold snowpack—is compromised, as the complete mixing assumption excludes the possibility of rapid transmission (vertically or laterally) of water through snowpacks which has been observed in experimental work [*Eiriksson et al*., 2013; *Juras et al*., 2016; *Evans et al*., 2016]. If a rain‐on‐snow event is intensive enough for water to percolate with little isotopic interaction with the snowpack, using complete mixing, as it is implemented in the model, would leave the snowpack biased toward the precipitation value (typically more enriched than snow) and the simulated runoff from the snowpack would be too depleted (assuming relatively enriched rainfall). This could be addressed with a partial mixing routine for rain‐on‐snow events building on isotope exchange experiments such as in *Juras et al*. [2016]. Based on their work the preferential flow component would need to be more prevalent for cold midwinter snowpacks than ripe snowpacks during the overall melt period.

The simulated enrichment for both sublimation and melting does not differentiate between equilibrium and kinetic fractionation, which are well documented processes in the literature [*O'Neil*, 1968; *Moser and Stichler*, 1974; *Gat and Gonfiantini*, 1981; *Gustafson et al*., 2010]. Rather the model lumps these processes into fractionation offset parameters, with the purpose of capturing the combined result of the two fractionation processes, circumventing the need to represent them explicitly. Parameterization of this offset can be kept in physically meaningful boundaries obtained from theoretical equilibrium fractionation factors [*Gat and Gonfiantini*, 1981; *Ellehoj et al*., 2013]. For sublimation, the limit for parameter *E*_*frac*_ (15‰) could be set higher, because the theoretical equilibrium fractionation factor decreases in cold air temperatures (approximately 20‰ in −30°C) [*Ellehoj et al*., 2013]. Furthermore, kinetic evaporation processes would lead to additional enrichment, which could be represented by allowing higher values for *E*_*frac*_. The kinetic isotope fractionation is enhanced in high temperature and low relative humidity, but environments with seasonal snowpacks typically exhibit low air temperature and high humidity over winter (see Figure 2) leading to less kinetic sublimation fractionation, as suggested by *Earman et al*. [2006].

In the model calibration presented here, the *E*_*frac*_ parameter shows a tendency to produce best simulations around the values of 5‰ at both sites, giving a hint of a generic parameter value which may be transferrable. Having the value closer to 0 than 15‰ is reasonable, because the evaporation in snow occurs from both liquid water retained in the snowpack and ice sublimation [*Wigmosta et al*., 1994], which are not explicitly separated in our model. However, evaporation from the liquid phase has a lower equilibrium fractionation factor of 9.8‰ [*Gat and Gonfiantini*, 1981] and would therefore lead to less bulk enrichment of the snowpack. Perhaps even more importantly, field and laboratory experiments, *Earman et al*. [2006] demonstrated that in addition to sublimation fractionation, snow undergoes ambient isotopic exchange with the atmosphere. *Earman et al*. [2006] showed how the theoretically calculated sublimation fractionation (equilibrium and kinetic) according to *Gat and Gonfiantini* [1981] would cause much greater snow enrichment than observed in their experiments. Our fractionation parameterization implicitly incorporates the ambient isotopic exchange over the winter, allowing the fractionation effects to be less than the theoretical, as suggested by empirical evidence in *Earman et al*. [2006], and as seen in our simulations where the behavioral range for the *E*_*frac*_ parameter is consistently below 15‰ (Figure 5).

The fractionation parameter for snowmelt *M*_*frac*_ appears to be less identifiable in the calibration. *Feng et al*. [2002] show how the melt rate affects the intensity of fractionation, with higher fractionation occurring during lower melt rates. Our approach relates the melt fractionation to melt history rather than melt rates, which is something that could be tested and modified if needed. In addition, we specified threshold constraints in the model that excluded snowmelt fractionation for shallow snowpacks (SWE < 10 mm) and low runoff rates (<2 mm d^−1^) based on model testing and theoretical considerations (see equation (4)). With more temporally continuous snowmelt lysimeter data sets for model testing [as in *Taylor et al*., 2001], these thresholds could be included as calibration parameters. The minimum snowpack SWE constraint was also useful in creating numerical stability for the isotope solution in shallow snowpacks.

Our spatially distributed process‐based formulation allows the snowmelt to occur at different rates and onset times in different parts of the catchment (Figure 3), depending on canopy cover [*Varhola et al*., 2010], aspect [*Carey and Quinton*, 2004], and altitude [*Blöschl et al*., 1991]. This brings the possibility of simulating variable source areas producing snowmelt runoff, which may be crucial for providing suitable input for tracer‐aided hydrological studies [*Laudon et al*., 2002; *Schmieder et al*., 2016]. However, in this work, we do not make an attempt to extend the simulations to study how the snowmelt water is stored, mixed and transported in the landscape. An example how the developed model can be coupled with a spatially distributed tracer‐aided rainfall‐runoff model to successfully estimate storage, mixing and age in the landscape is given in a parallel study [*Ala‐aho et al*., 2017].

Our process‐based snow module to simulate SWE is what one could call “minimalistic” in terms of the processes included and the complexity with which they are conceptualized. One major missing model component is wind redistribution of snow that could make our approach unsuitable especially for complex mountainous terrain [*Blöschl et al*., 1991] or windswept tundra environments [*Bowling et al*., 2004]. Empirical routines for wind redistribution of snowfall [*Winstral et al*., 2002; *Broxton et al*., 2015], where snow is redistributed before settling on the existing snowpack, could be readily be implemented in the model. More physically based blowing snow routines, in which the settled snowpack is redistributed [*Essery et al*., 1999; *Bowling et al*., 2004], would be more challenging to incorporate due to our simplifying assumption for complete mixing of isotopes in the snowpack. Incorporating physically based routines would benefit from a layered structure in the snow isotope model, because blowing snow redistribution would affect primarily the snow surface, i.e., the most recently fallen layer, bearing a specific isotope signature. Furthermore, our snow accumulation/melt model realism could be improved for ground, intercepted and blowing snow sublimation, evolution of snow albedo, canopy transmissivity and sheltering, and snow temperature [*Jordan*, 1991; *Tarboton and Luce*, 1996; *Pomeroy et al*., 1998; *Essery et al*., 1999; *Lehning et al*., 2002; *Liston and Elder*, 2006; *Pomeroy et al*., 2007; *Broxton et al*., 2015], with the trade‐off of increasing model parameterization. However, our objective here was to produce a spatially distributed process‐based based snow model [as in *Walter et al*., 2005] to serve as an adequate basis for spatially variable snow accumulation and melt. Our good success in simulating SWE at the field sites (Figure 4) shows that this aim was satisfied for the given sites, but modifications may be required when adapting the model to different environments.

As for any numerical environmental modeling, our approach is based on the need to calibrate the fractionation parameters against field observations. Here we use samples of snowmelt, but in a parallel study [*Ala‐aho et al*., 2017], we show how the isotopic composition of streamflow can be used to inform the snow isotope model calibration. In addition, cored samples from the bulk isotopic composition of snowpack could be used. It is unlikely that with its simplistic fractionation parameterization our model will be able to reproduce the detailed snowmelt runoff isotopic composition at point scale with the details presented in *Claassen and Downey* [1995], *Taylor et al*. [2001], and *Feng et al*. [2002]. However, the model retains the skill to capture trends in variability at the landscape scale that seems promising from both the theoretical experiment (Figure 3) and field proof‐of‐concept empirical field data (Figure 4). To fully test the spatially distributed model realism, a comprehensive field study focusing the sampling in different landscape locations and under different canopy covers is warranted. The sampling scheme will need enough replicates in each location to uncover the general trends from typically extensive point‐scale variability.

## 7 Conclusions

We present a novel parsimonious modeling approach to simulate for the first time the spatially and temporally variable isotopic composition of snowmelt at the landscape scale. The resulting isotope ratios of snowmelt can be used in increasingly common tracer‐aided modeling studies to better understand the integrated nature of flow, mixing, and transport processes in snow‐influenced regions. The model‐based estimates for snowmelt isotopes can serve as input for tools such as end‐member mixing analysis, hydrograph separation, groundwater recharge source estimation, or tracer‐aided hydrological model applications.

Poor estimates for the snowmelt isotope input signal have been identified as a major source of uncertainty for the above techniques in northern snow‐influenced environments. Our new model shows promise in producing an improved spatiotemporal estimate for snowmelt isotopes, which leaves the subsequent numerical technique, whatever it may be, with fewer degrees of freedom to constrain the water flow, mixing, and transport processes in the landscape. Furthermore, the model introduces a new methodology for simulating the sublimation enrichment of heavy isotopes in canopy‐intercepted snow. Isotope alterations caused by canopy interception can be important to account for in trace‐aided techniques where the hydrological partitioning of vegetation canopies is of interest.

Our model is relatively simplistic, and in its current form may not transfer well to some snow environments; such as mountainous or tundra areas where wind redistribution is important, or rain‐snow transition zones where rain‐on‐snow events are common. On the other hand, a major advantage of the parsimonious isotope routines is that they can be effortlessly incorporated to any snow accumulation and melt model that explicitly accounts for snow sublimation. Therefore, the isotope routines can readily be applied, tested, and further developed using existing snow models and isotope data sets in contrasting environmental settings. Our work shows considerable promise in the model's plausible process representation in a theoretical simulation exercise, and an empirically based test in two field sites with contrasting snow conditions, but further model testing in different snow environments is needed.

## Supporting information

Supporting Information S1

## References

[wrcr22746-bib-0001] Ala‐aho, P. , D. Tetzlaff , J. P. McNamara , H. Laudon , and C. Soulsby (2017), Using isotopes to constrain water flux and age estimates in snow‐influenced catchments using the STARR (Spatially distributed Tracer‐Aided Rainfall‐Runoff) model, Hydrol. Earth Syst. Sci. Discuss., doi:10.5194/hess-2017-106.

[wrcr22746-bib-0002] Anderson, E. A. (1968), Development and testing of snow pack energy balance equations, Water Resour. Res., 4, 19–37, doi:10.1029/WR004i001p00019.

[wrcr22746-bib-0003] Barnett, T. P. , J. C. Adam , and D. P. Lettenmaier (2005), Potential impacts of a warming climate on water availability in snow‐dominated regions, Nature, 438, 303–309, doi:10.1038/nature04141. 16292301 10.1038/nature04141

[wrcr22746-bib-0004] Berman, E. S. , M. Gupta , C. Gabrielli , T. Garland , and J. J. McDonnell (2009), High‐frequency field‐deployable isotope analyzer for hydrological applications, Water Resour. Res., 45, W10201, doi:10.1029/2009WR008265.

[wrcr22746-bib-0005] Beven, K. , and J. Freer (2001), Equifinality, data assimilation, and uncertainty estimation in mechanistic modelling of complex environmental systems using the GLUE methodology, J. Hydrol., 249, 11–29, doi:10.1016/S0022-1694(01)00421-8.

[wrcr22746-bib-0006] Birkel, C. , and C. Soulsby (2015), Advancing tracer‐aided rainfall‐runoff modelling: A review of progress, problems and unrealised potential, Hydrol. Processes, 29, 5227–5240, doi:10.1002/hyp.10594.

[wrcr22746-bib-0007] Blöschl, G. , R. Kirnbauer , and D. Gutknecht (1991), Distributed snowmelt simulations in an alpine catchment: 1. Model evaluation on the basis of snow cover patterns, Water Resour. Res., 27, 3171–3179, doi:10.1029/91WR02250.

[wrcr22746-bib-0008] Bowling, L. , J. Pomeroy , and D. Lettenmaier (2004), Parameterization of blowing‐snow sublimation in a macroscale hydrology model, J. Hydrometeorol., 5, 745–762, doi:10.1175/1525-7541(2004)005<0745:POBSIA>2.0.CO;2.

[wrcr22746-bib-0009] Broxton, P. , A. Harpold , J. Biederman , P. A. Troch , N. Molotch , and P. D. Brooks (2015), Quantifying the effects of vegetation structure on snow accumulation and ablation in mixed‐conifer forests, Ecohydrology, 8, 1073–1094, doi:10.1002/eco.1565.

[wrcr22746-bib-0010] Carey, S. , and W. Quinton (2004), Evaluating snowmelt runoff generation in a discontinuous permafrost catchment using stable isotope, hydrochemical and hydrometric data, Hydrol. Res., 35, 309–324.

[wrcr22746-bib-0011] Claassen, H. , and J. Downey (1995), A model for deuterium and oxygen 18 isotope changes during evergreen interception of snowfall, Water Resour. Res., 31, 601–618, doi:10.1029/94WR01995.

[wrcr22746-bib-0012] Dahlke, H. E. , and S. W. Lyon (2013), Early melt season snowpack isotopic evolution in the Tarfala valley, northern Sweden, Ann. Glaciol., 54, 149–156, doi:10.3189/2013AoG62A232.

[wrcr22746-bib-0013] Delavau, C. , T. Stadnyk , and T. Holmes (2017), Examining the impacts of precipitation isotope input (^18^O_ppt_) on distributed, tracer‐aided hydrological modelling, Hydrol. Earth Syst. Sci., 21, 2595–2614, doi:10.5194/hess-21-2595-2017.

[wrcr22746-bib-0014] Dietermann, N. , and M. Weiler (2013), Spatial distribution of stable water isotopes in alpine snow cover, Hydrol. Earth Syst. Sci., 17, 2657–2668, doi:10.5194/hess-17-2657-2013.

[wrcr22746-bib-0015] Earman, S. , A. R. Campbell , F. M. Phillips , and B. D. Newman (2006), Isotopic exchange between snow and atmospheric water vapor: Estimation of the snowmelt component of groundwater recharge in the southwestern United States, J. Geophys. Res., 111, D09302, doi:10.1029/2005JD006470.

[wrcr22746-bib-0016] Eiriksson, D. , M. Whitson , C. H. Luce , H. P. Marshall , J. Bradford , S. G. Benner , T. Black , H. Hetrick , and J. P. McNamara (2013), An evaluation of the hydrologic relevance of lateral flow in snow at hillslope and catchment scales, Hydrol. Processes, 27, 640–654, doi:10.1002/hyp.9666.

[wrcr22746-bib-0017] Ellehoj, M. , H. C. Steen‐Larsen , S. J. Johnsen , and M. B. Madsen (2013), Ice‐vapor equilibrium fractionation factor of hydrogen and oxygen isotopes: Experimental investigations and implications for stable water isotope studies, Rapid Commun. Mass Spectrom., 27, 2149–2158, doi:10.1002/rcm.6668. 23996388 10.1002/rcm.6668

[wrcr22746-bib-0018] Eskelinen, R. , A. Ronkanen , H. Marttila , E. Isokangas , and B. Kløve (2016), Effects of soil frost on snowmelt runoff generation and surface water quality in drained peatlands, Boreal Environ. Res., 21, 556–570.

[wrcr22746-bib-0019] ESRI (2011), *ARcGIS Desktop: Release 10*, Environmental Systems Research Institute, Redlands, Calif.

[wrcr22746-bib-0020] Essery, R. , L. Li , and J. Pomeroy (1999), A distributed model of blowing snow over complex terrain, Hydrol. Processes, 13, 2423–2438.

[wrcr22746-bib-0021] Evans, S. L. , A. N. Flores , A. Heilig , M. J. Kohn , H. Marshall , and J. P. McNamara (2016), Isotopic evidence for lateral flow and diffusive transport, but not sublimation, in a sloped seasonal snowpack, Idaho, USA, Geophys. Res. Lett., 43, 3298–3306, doi:10.1002/2015GL067605.

[wrcr22746-bib-0022] Fekete, B. M. , J. J. Gibson , P. Aggarwal , and C. J. Vörösmarty (2006), Application of isotope tracers in continental scale hydrological modeling, J. Hydrol., 330, 444–456, doi:10.1016/j.jhydrol.2006.04.029.

[wrcr22746-bib-0023] Feng, X. , S. Taylor , C. E. Renshaw , and J. W. Kirchner (2002), Isotopic evolution of snowmelt: 1. A physically based one‐dimensional model, Water Resour. Res., 38(10), 1217, doi:10.1029/2001WR000814.

[wrcr22746-bib-0024] Gat, J. R. , and R. Gonfiantini (1981), Stable isotope hydrology. Deuterium and oxygen‐18 in the water cycle, *Tech. Rep. Ser. 210*, IAEA, Va.

[wrcr22746-bib-0025] Gupta, H. V. , H. Kling , K. K. Yilmaz , and G. F. Martinez (2009), Decomposition of the mean squared error and NSE performance criteria: Implications for improving hydrological modelling, J. Hydrol., 377, 80–91, doi:10.1016/j.jhydrol.2009.08.003.

[wrcr22746-bib-0026] Gustafson, J. R. , P. D. Brooks , N. Molotch , and W. Veatch (2010), Estimating snow sublimation using natural chemical and isotopic tracers across a gradient of solar radiation, Water Resour. Res., 46, W12511, doi:10.1029/2009WR009060.

[wrcr22746-bib-0027] Hedstrom, N. , and J. Pomeroy (1998), Measurements and modelling of snow interception in the boreal forest, Hydrol. Processes, 12, 1611–1625, doi:10.1002/(SICI)1099-1085(199812)12:15<2317::AID-HYP799>3.0.CO;2-X.

[wrcr22746-bib-0028] Homan, J. W. , C. H. Luce , J. P. McNamara , and N. F. Glenn (2011), Improvement of distributed snowmelt energy balance modeling with MODIS‐based NDSI‐derived fractional snow‐covered area data, Hydrol. Processes, 25, 650–660, doi:10.1002/hyp.7857.

[wrcr22746-bib-0029] Jasechko, S. , L. I. Wassenaar , and B. Mayer (2017), Isotopic evidence for widespread cold‐season‐biased groundwater recharge and young streamflow across central Canada, Hydrol. Processes, doi:10.1002/hyp.11175.

[wrcr22746-bib-0030] Jordan, R. (1991), A one‐dimensional temperature model for a snow cover: Technical documentation for SNTHERM.89, in *A One‐Dimensional Temperature Model for a Snow Cover: Technical Documentation for SNTHERM.89*, Cold Regions Research and Engineering Lab, Hanover, N. H.

[wrcr22746-bib-0031] Juras, R. , S. Würzer , J. Pavlasek , and T. Jonas (2016), Rainwater propagation through snowpack during rain‐on‐snow events under different snow condition, Hydrol. Earth Syst. Sci. Discuss., doi:0.5194/hess-2016-612.

[wrcr22746-bib-0032] Karlsen, R. H. , T. Grabs , K. Bishop , I. Buffam , H. Laudon , and J. Seibert (2016), Landscape controls on spatiotemporal discharge variability in a boreal catchment, Water Resour. Res., 52, 6541–6556, doi:10.1002/2016WR019186.

[wrcr22746-bib-0033] Karssenberg, D. , O. Schmitz , P. Salamon , K. de Jong , and M. F. P. Bierkens (2010), A software framework for construction of process‐based stochastic spatio‐temporal models and data assimilation, Environ. Modell. Software, 25, 489–502, doi:10.1016/j.envsoft.2009.10.004.

[wrcr22746-bib-0034] Kelleners, T. , D. Chandler , J. P. McNamara , M. M. Gribb , and M. Seyfried (2010), Modeling runoff generation in a small snow‐dominated mountainous catchment, Vadose Zone J., 9, 517–527, doi:10.2136/vzj2009.0033.

[wrcr22746-bib-0035] Kendall, C. , and J. J. McDonnell (1998), Isotope Tracers in Catchment Hydrology, Elsevier, Amsterdam.

[wrcr22746-bib-0036] Kirchner, J. W. (2006), Getting the right answers for the right reasons: Linking measurements, analyses, and models to advance the science of hydrology, Water Resour. Res., 42, W03S04, doi:10.1029/2005WR004362.

[wrcr22746-bib-0037] Koeniger, P. , J. A. Hubbart , T. Link , and J. D. Marshall (2008), Isotopic variation of snow cover and streamflow in response to changes in canopy structure in a snow‐dominated mountain catchment, Hydrol. Processes, 22, 557–566, doi:10.1002/hyp.6967.

[wrcr22746-bib-0038] Kormos, P. M. (2005), Accounting for time and space variations of δ^18^ in a snowmelt isotopic hydrograph separation in the Boise front, *Rep. 916*, pp. 1–57, Boise State Univ., Boise, Idaho.

[wrcr22746-bib-0039] Kormos, P. R. , D. Marks , J. P. McNamara , H. Marshall , A. Winstral , and A. N. Flores (2014), Snow distribution, melt and surface water inputs to the soil in the mountain rain–snow transition zone, J. Hydrol., 519, 190–204, doi:10.1016/j.jhydrol.2014.06.051.

[wrcr22746-bib-0040] Laudon, H. , and M. O. Löfvenius (2016), Adding snow to the picture—Providing complementary winter precipitation data to the Krycklan catchment study database, Hydrol. Processes, 30, 2413–2416, doi:10.1002/hyp.10753.

[wrcr22746-bib-0041] Laudon, H. , H. F. Hemond , R. Krouse , and K. H. Bishop (2002), Oxygen 18 fractionation during snowmelt: Implications for spring flood hydrograph separation, Water Resour. Res., 38, 1258, doi:10.1029/2002WR001510.

[wrcr22746-bib-0042] Laudon, H. , J. Seibert , S. Köhler , and K. Bishop (2004), Hydrological flow paths during snowmelt: Congruence between hydrometric measurements and oxygen 18 in meltwater, soil water, and runoff, Water Resour. Res., 40, W03102, doi:10.1029/2003WR002455.

[wrcr22746-bib-0043] Laudon, H. , I. Taberman , A. Ågren , M. Futter , M. Ottosson‐Löfvenius , and K. Bishop (2013), The Krycklan Catchment Study—A flagship infrastructure for hydrology, biogeochemistry, and climate research in the boreal landscape, Water Resour. Res., 49, 7154–7158, doi:10.1002/wrcr.20520.

[wrcr22746-bib-0044] Lehning, M. , P. Bartelt , B. Brown , and C. Fierz (2002), A physical SNOWPACK model for the Swiss avalanche warning: Part III: Meteorological forcing, thin layer formation and evaluation, Cold Reg. Sci. Technol., 35, 169–184, doi:10.1016/S0165-232X(02)00072-1.

[wrcr22746-bib-0045] Liston, G. E. , and K. Elder (2006), A distributed snow‐evolution modeling system (SnowModel), J. Hydrometeorol., 7, 1259–1276, doi:10.1175/JHM548.1.

[wrcr22746-bib-0046] Lundberg, A. , and S. Halldin (2001), Snow interception evaporation. Review of measurement techniques, processes, and models, Theor. Appl. Clim., 70, 117–133, doi:10.1007/s007040170010.

[wrcr22746-bib-0047] Lundberg, A. , and H. Koivusalo (2003), Estimating winter evaporation in boreal forests with operational snow course data, Hydrol. Processes, 17, 1479–1493, doi:10.1002/hyp.1179.

[wrcr22746-bib-0048] Lyon, S. W. , H. Laudon , J. Seibert , M. Mörth , D. Tetzlaff , and K. H. Bishop (2010), Controls on snowmelt water mean transit times in northern boreal catchments, Hydrol. Processes, 24, 1672–1684, doi:10.1002/hyp.7577.

[wrcr22746-bib-0049] Marks, D. , and A. Winstral (2001), Comparison of snow deposition, the snow cover energy balance, and snowmelt at two sites in a semiarid mountain basin, J. Hydrometeorol., 2, 213–227, doi:10.3189/172756401781819751.

[wrcr22746-bib-0050] Moser, H. , and W. Stichler (1974), Deuterium and oxygen‐18 contents as an index of the properties of snow covers, Int. Assoc. Hydrol. Sci. Publ., 114, 122–135.

[wrcr22746-bib-1051] National Climatic Data Center (2016), NESDIS, NOAA, U.S. Department of Commerce, Bogus Basin, Boise.

[wrcr22746-bib-0051] O'Neil, J. R. (1968), Hydrogen and oxygen isotope fractionation between ice and water, J. Phys. Chem., 72, 3683–3684.

[wrcr22746-bib-0052] Peralta‐Tapia, A. , C. Soulsby , D. Tetzlaff , R. Sponseller , K. Bishop , and H. Laudon (2016), Hydroclimatic influences on non‐stationary transit time distributions in a boreal headwater catchment, J. Hydrol., 543, 7–16, doi:10.1016/j.jhydrol.2016.01.079.

[wrcr22746-bib-0053] Pomeroy, J. , J. Parviainen , N. Hedstrom , and D. Gray (1998), Coupled modelling of forest snow interception and sublimation, Hydrol. Processes, 12, 2317–2337.

[wrcr22746-bib-0054] Pomeroy, J. , D. Gray , N. Hedstrom , and J. Janowicz (2002), Physically based estimation of seasonal snow accumulation in the boreal forest, paper presented at the 59th Eastern Snow Conference.

[wrcr22746-bib-0055] Pomeroy, J. , D. Gray , T. Brown , N. Hedstrom , W. Quinton , R. Granger , and S. Carey (2007), The cold regions hydrological model: A platform for basing process representation and model structure on physical evidence, Hydrol. Processes, 21, 2650–2667, doi:10.1002/hyp.6787.

[wrcr22746-bib-0056] Rasmus, S. , D. Gustafsson , R. Lundell , and T. Saarinen (2016), Observations and snow model simulations of winter energy balance terms within and between different coniferous forests in Southern Boreal Finland, Hydrol. Res., 47, 201–216, doi:10.2166/nh.2015.177.

[wrcr22746-bib-0057] Rinehart, A. J. , E. R. Vivoni , and P. D. Brooks (2008), Effects of vegetation, albedo, and solar radiation sheltering on the distribution of snow in the Valles Caldera, New Mexico, Ecohydrology, 1, 253–270, doi:10.1002/eco.26.

[wrcr22746-bib-0058] Rodhe, A. (1981), Spring flood meltwater or groundwater?, Hydrol. Res., 12, 21–30.

[wrcr22746-bib-0059] Schmieder, J. , F. Hanzer , T. Marke , J. Garvelmann , M. Warscher , H. Kunstmann , and U. Strasser (2016), The importance of spatio‐temporal snowmelt variability for isotopic hydrograph separation in a high‐elevation catchment, Hydrol. Earth Syst. Sci. Discuss., 2016, 1–27, doi:10.5194/hess-2016-128.

[wrcr22746-bib-0060] Shanley, J. B. , C. Kendall , M. R. Albert , and J. P. Hardy (1995), Chemical and isotopic evolution of a layered eastern US snowpack and its relation to stream‐water composition, *IAHS Publ. Ser. Proc. Rep. Int. Assoc. Hydrol. Sci*, 228, 329–338.

[wrcr22746-bib-0061] Sklash, M. G. , and R. N. Farvolden (1979), The role of groundwater in storm runoff, Dev. Water Sci., 12, 45–65, doi:10.1016/S0167-5648(09)70009-7.

[wrcr22746-bib-0062] Smith, A. , C. Welch , and T. Stadnyk (2016), Assessment of a lumped coupled flow‐isotope model in data scarce Boreal catchments, Hydrol. Process., 30, 3871–3884, doi:10.1002/hyp.10835.

[wrcr22746-bib-0063] Soulsby, C. , R. Malcolm , R. Helliwell , R. C. Ferrier , and A. Jenkins (2000), Isotope hydrology of the Allt a' Mharcaidh catchment, Cairngorms, Scotland: Implications for hydrological pathways and residence times, Hydrol. Processes, 14, 747–762, doi:10.1002/(SICI)1099-1085(200003)14:4<747::AID-HYP970>3.0.CO;2-0.

[wrcr22746-bib-0064] Soulsby, C. , C. Birkel , J. Geris , J. Dick , C. Tunaley , and D. Tetzlaff (2015), Stream water age distributions controlled by storage dynamics and nonlinear hydrologic connectivity: Modeling with high‐resolution isotope data, Water Resour. Res., 51, 7759–7776, doi:10.1002/2015WR017888. 27478255 10.1002/2015WR017888 PMC4949550

[wrcr22746-bib-0065] Stadnyk, T. A. , C. Delavau , N. Kouwen , and T. W. D. Edwards (2013), Towards hydrological model calibration and validation: Simulation of stable water isotopes using the isoWATFLOOD model, Hydrol. Processes, 27, 3791–3810, doi:10.1002/hyp.9695.

[wrcr22746-bib-0066] Stichler, W. , W. Rauert , and J. Martinec (1981), Environmental isotope studies of an alpine snowpack, Nord. Hydrol., 12, 297–308.

[wrcr22746-bib-0067] Tappa, D. J. , M. J. Kohn , J. P. McNamara , S. G. Benner , and A. N. Flores (2016), Isotopic composition of precipitation in a topographically steep, seasonally snow‐dominated watershed and implications of variations from the Global Meteoric Water Line, Hydrol. Processes, 30, 4582–4592, doi:10.1002/hyp.10940.

[wrcr22746-bib-0068] Tarboton, D. G. , and C. H. Luce (1996), *Utah Energy Balance Snow Accumulation and Melt Model (UEB)*, Utah Water Res. Lab. and U.S. Dep. of Agric. For. Serv.

[wrcr22746-bib-0069] Taylor, S. , X. Feng , J. W. Kirchner , R. Osterhuber , B. Klaue , and C. E. Renshaw (2001), Isotopic evolution of a seasonal snowpack and its melt, Water Resour. Res., 37, 759–769, doi:10.1029/2000WR900341.

[wrcr22746-bib-0070] Taylor, S. , X. Feng , M. Williams , and J. McNamara (2002), How isotopic fractionation of snowmelt affects hydrograph separation, Hydrol. Processes, 16, 3683–3690, doi:10.1002/hyp.1232.

[wrcr22746-bib-0071] Tetzlaff, D. , J. Buttle , S. K. Carey , K. McGuire , H. Laudon , and C. Soulsby (2015), Tracer‐based assessment of flow paths, storage and runoff generation in northern catchments: A review, Hydrol. Processes, 29, 3475–3490, doi:10.1002/hyp.10412.

[wrcr22746-bib-0072] Unnikrishna, P. V. , J. J. McDonnell , and C. Kendall (2002), Isotope variations in a Sierra Nevada snowpack and their relation to meltwater, J. Hydrol., 260, 38–57, doi:10.1016/S0022-1694(01)00596-0.

[wrcr22746-bib-0073] Varhola, A. , N. C. Coops , M. Weiler , and R. D. Moore (2010), Forest canopy effects on snow accumulation and ablation: An integrative review of empirical results, J. Hydrol., 392, 219–233, doi:10.1016/j.jhydrol.2010.08.009.

[wrcr22746-bib-0074] Walter, M. T. , E. S. Brooks , D. K. McCool , L. G. King , M. Molnau , and J. Boll (2005), Process‐based snowmelt modeling: Does it require more input data than temperature‐index modeling?, J. Hydrol., 300, 65–75, doi:10.1016/j.jhydrol.2004.05.002.

[wrcr22746-bib-0075] Wigmosta, M. S. , L. W. Vail , and D. P. Lettenmaier (1994), A distributed hydrology‐vegetation model for complex terrain, Water Resour. Res., 30, 1665–1679, doi:10.1029/94WR00436.

[wrcr22746-bib-0076] Winstral, A. , K. Elder , and R. E. Davis (2002), Spatial snow modeling of wind‐redistributed snow using terrain‐based parameters, J. Hydrometeorol., 3, 524–538, doi:10.1175/1525-7541(2002)003<0524:SSMOWR>2.0.CO;2.

[wrcr22746-bib-0077] Yang, D. , B. E. Goodison , J. R. Metcalfe , V. S. Golubev , R. Bates , T. Pangburn , and C. L. Hanson (1998), Accuracy of NWS 8″ standard nonrecording precipitation gauge: Results and application of WMO intercomparison, J. Atmos. Oceanic. Technol., 15, 54–68, doi:10.1175/1520-0426(1998)015<0054:AONSNP>2.0.CO;2.

