# Supplementary material for: Modeling the isotopic evolution of snowpack and snowmelt: Testing a spatially distributed parsimonious approach
Source: Water Resour Res. 2017 Jul 20;53(7):5813–30. doi: 10.1002/2017WR020650 (PMC5601190; doi:10.1002/2017WR020650)
Supplement: Supplementary file 1 — Supporting Information S1 [file WRCR-53-5813-s001.docx]

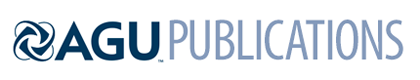


*Water Resources Research*

Supporting Information for

**Modeling the isotopic evolution of snowpack and snowmelt: testing a spatially distributed parsimonious approach**

Pertti Ala-aho^1^, Doerthe Tetzlaff^1^, James P. McNamara^2^, Hjalmar Laudon^3^, Patrick Kormos^4^, Chris Soulsby^1^

^1^Northern Rivers Institute, School of Geosciences, University of Aberdeen, UK, AB24 3UF

^2^Department of Geosciences, Boise State University, Boise, ID 83725, USA

^3^Department of Forest, Ecology and Management, Swedish University of Agricultural Sciences, 90183 Umeå, Sweden.

^4^United States Department of Agriculture, Agricultural Research Service, Boise, ID 83712, USA

**Contents of this file**

Tables S1 and S2

**Introduction**

- The supplementary information S1 contains the equations and related references used to simulate spatially distributed snow interception, accumulation and melt
- Table S2 contains the snow lysimeter data visualized in Figure 4. Table S2, like the Figure 4, reports the average and standard deviation isotope ratio in δ^18^O sampled with multiple snowmelt lysimeters (typically 9 per day for Krycklan and 5 per day for Bogus).

| $\boldsymbol{P}_{\boldsymbol{q}}\boldsymbol{=}\begin{matrix} \boldsymbol{1} & \boldsymbol{T}_{\boldsymbol{a}}\boldsymbol{<}\boldsymbol{TT}_{\boldsymbol{low}} \\ \frac{\boldsymbol{TT}_{\boldsymbol{high}}\boldsymbol{-}\boldsymbol{T}_{\boldsymbol{a}}}{\boldsymbol{TT}_{\boldsymbol{high}}\boldsymbol{-}\boldsymbol{TT}_{\boldsymbol{low}}} & {{\boldsymbol{T}\boldsymbol{T}}_{\boldsymbol{low}}\boldsymbol{<T}}_{\boldsymbol{a}}\boldsymbol{<}\boldsymbol{TT}_{\boldsymbol{high}} \\ \boldsymbol{0} & \boldsymbol{T}_{\boldsymbol{a}}\boldsymbol{>}\boldsymbol{TT}_{\boldsymbol{high}} \end{matrix}$ (S1) | Thermal quality of precipitation P_q_;  T_a_ [⁰C]: air temperature; TT_low_ [⁰C]: threshold temperature below which all precipitation is snow and TT_high_ [⁰C]: threshold temperature above which all precipitation is liquid |
| --- | --- |
| $\boldsymbol{c}_{\boldsymbol{snow}}\boldsymbol{=}\begin{matrix} \left( \frac{\mathbf{exp}\boldsymbol{(4.606-0.036*}\boldsymbol{W}_{\boldsymbol{can}}^{\boldsymbol{1.75}}\boldsymbol{)}}{\boldsymbol{100}} \right)^{\boldsymbol{-1}} & \boldsymbol{T}_{\boldsymbol{a}}\boldsymbol{<0} \\ \left( \frac{\boldsymbol{101.04-5.62*}\boldsymbol{W}_{\boldsymbol{can}}}{\boldsymbol{100}} \right)^{\boldsymbol{-1}} & \boldsymbol{T}_{\boldsymbol{a}}\boldsymbol{>0} \end{matrix}$ (S2) | Correction coefficient for under catch of snow precipitation due to wind c_snow_ [*Yang et al.,* 1998]:  W_can_ [m s^-1^]: wind speed within tree canopy |
| $\boldsymbol{W}_{\boldsymbol{can}}\boldsymbol{=W*(1-}\left( \boldsymbol{0.8*}\boldsymbol{c}_{\boldsymbol{cov}} \right)\boldsymbol{)}$ (S3) | W_can_ [*Tarboton and Luce,* 1996];  W [m s^-1^]: measured wind speed and c_cov_ [-]: canopy closure fraction estimated |
| $\boldsymbol{c}_{\boldsymbol{cov}}\boldsymbol{=0.29*}\ln\left( \boldsymbol{LAI} \right)\boldsymbol{+0.55}$ (S4) | c_cov_ [*Pomeroy et al.,* 2002]; LAI [-]: canopy leaf area index. |
| $\boldsymbol{c}_{\boldsymbol{snow}}\boldsymbol{=}\begin{matrix} {\boldsymbol{c}_{\boldsymbol{snow}}\boldsymbol{+c}}_{\boldsymbol{corr}} & \boldsymbol{c}_{\boldsymbol{snow}}\boldsymbol{<1.5} \\ \boldsymbol{c}_{\boldsymbol{snow}} & \boldsymbol{c}_{\boldsymbol{snow}}\boldsymbol{>1.5} \end{matrix}$ (S5) | An empirical correlation coefficient c_corr_ was added to account for other than wind under catch for low c_snow_ values: |
| $\boldsymbol{P}_{\boldsymbol{liq}}\boldsymbol{=P*(1-}\boldsymbol{P}_{\boldsymbol{q}}\boldsymbol{)}$ (S6) | Liquid precipitation P_liq_ [m d^-1^]; P [m d^-1^]: measured precipitation |
| $\boldsymbol{P}_{\boldsymbol{ice}}\boldsymbol{=P*}\boldsymbol{P}_{\boldsymbol{q}}\boldsymbol{*}\boldsymbol{c}_{\boldsymbol{snow}}$ (S7) | Snow precipitation P_ice_ [m d^-1^]; |
| $\boldsymbol{R}_{\boldsymbol{net}}\boldsymbol{=}\boldsymbol{R}_{\boldsymbol{s}}\left( \boldsymbol{1-a} \right)\boldsymbol{*(}\boldsymbol{\tau}_{\boldsymbol{0}}\boldsymbol{*}\boldsymbol{c}_{\boldsymbol{cov}}\boldsymbol{+}\left( \boldsymbol{1-}\boldsymbol{c}_{\boldsymbol{cov}} \right)\boldsymbol{)}$ (S8) | R_net_ [MJ m^-2^ d^-1^] adjusted net shortwave radiation [*Wigmosta et al.,* 1994]; Incoming shortwave radiation R_s_ [MJ m^-2^ d^-1^]; a [-] snow albedo; τ_0_ [-]: fraction of shortwave radiation transmitted by the canopy *. |
| * Radiation R_s_ is spatially distributed within the landscape to account for aspect, topographical shading and changing solar angle over the course of the year. First, we use ArcGIS package AreaSolarRadiation which calculates the incoming clear-sky radiation for a raster surface [*ESRI,* 2011]. Using the resulting raster, we calculate the ratio between all cells in the model domain and the cell receiving most radiation exposure, giving a ratio between [0-1] in all cells. This ratio is then multiplied by the measured incoming shortwave radiation, assuming that the exposure at the measurement location equates to the most exposed model cell. | |
| $\boldsymbol{a=}{\boldsymbol{0.94}^{\boldsymbol{d}_{\boldsymbol{ns}}}}^{\boldsymbol{0.58}}$ (S9) | Snow albedo [-] [*Wigmosta et al.,* 1994]; d_ns_ [-]: number days without snowfall. |
| $\begin{matrix} \boldsymbol{a=}\boldsymbol{a}^{\boldsymbol{a}_{\boldsymbol{pow}}} & \boldsymbol{,}\boldsymbol{d}_{\boldsymbol{ns}}\boldsymbol{>100} \end{matrix}$ (S10) | a_pow_ [-]: parameter accounting for the decline of the albedo in old snow |
| $\boldsymbol{\tau}_{\boldsymbol{0}}\boldsymbol{=}\mathbf{exp}\boldsymbol{(}\boldsymbol{-c}_{\boldsymbol{att}}\boldsymbol{*LAI)}$ (S11) | τ_0_ [-] [*Wigmosta et al.,* 1994]; Where c_att_ [-]: attenuation coefficient. |
| $\boldsymbol{L}_{\boldsymbol{net}}\boldsymbol{=}\boldsymbol{L}_{\boldsymbol{0}}\boldsymbol{*}\boldsymbol{c}_{\boldsymbol{cov}}\boldsymbol{+}\left( \boldsymbol{L}_{\boldsymbol{d}}\boldsymbol{*}\left( \boldsymbol{1-}\boldsymbol{c}_{\boldsymbol{cov}} \right) \right)\boldsymbol{-}\boldsymbol{L}_{\boldsymbol{s}}$ (S12) | Net longwave radiation L_net_ [MJ m^-2^ d^-1^]; L_0_ [MJ m^-2^ d^-1^]: long wave radiation emitted by the canopy |
| $\boldsymbol{L}_{\boldsymbol{0}}\boldsymbol{=}\boldsymbol{c}_{\boldsymbol{SB}}\boldsymbol{*}{\boldsymbol{(273.15+}\boldsymbol{T}_{\boldsymbol{a}}\boldsymbol{)}}^{\boldsymbol{4}}$ (S13) | L_0_ [MJ m^-2^ d^-1^]: c_SB_ is the Stefan-Boltzman constant 4.89 E-9 [MJ d^-1^ m^-2^ ⁰C^-4^]; assuming emissivity of unity. |
| $\boldsymbol{L}_{\boldsymbol{d}}\boldsymbol{=}\boldsymbol{e}_{\boldsymbol{air}}\boldsymbol{*}\boldsymbol{c}_{\boldsymbol{SB}}{\boldsymbol{(273.15+}\boldsymbol{T}_{\boldsymbol{a}}\boldsymbol{)}}^{\boldsymbol{4}}$ (S14) | L_d_ [MJ m^-2^ d^-1^]: atmospheric longwave radiation; e_air_ [-]: atmosphere emissivity |
| $\boldsymbol{e}_{\boldsymbol{air}}\boldsymbol{=}\begin{matrix} \left( \boldsymbol{0.72+0.005*}\boldsymbol{T}_{\boldsymbol{a}} \right)\boldsymbol{*}\left( \boldsymbol{1-0.84} \right)\boldsymbol{+0.84} & \boldsymbol{P>}\boldsymbol{P}_{\boldsymbol{thres}} \\ \boldsymbol{0.72+0.005*}\boldsymbol{T}_{\boldsymbol{a}} & \boldsymbol{P\leq}\boldsymbol{P}_{\boldsymbol{thres}} \end{matrix}$  (S15) | e_air_ is estimated differently for clear and cloudy days with a threshold daily precipitation P_thres_ differentiating clear and cloudy days [*Walter et al.,* 2005] |
| $\boldsymbol{L}_{\boldsymbol{s}}\boldsymbol{=0.97*}\boldsymbol{c}_{\boldsymbol{SB}}{\boldsymbol{(273.15+}\boldsymbol{T}_{\boldsymbol{sn}}\boldsymbol{)}}^{\boldsymbol{4}}$ (S16) | Longwave radiation emitted by the snow L_s_ [MJ m^-2^ d^-1^]; T_sn_ [⁰C ]: simulated snow temperature. |
| $\boldsymbol{Q}_{\boldsymbol{P}}\boldsymbol{=}\boldsymbol{\rho}_{\boldsymbol{w}}\boldsymbol{*}\boldsymbol{C}_{\boldsymbol{w}}\boldsymbol{*}\boldsymbol{T}_{\boldsymbol{a}}\boldsymbol{*(}\boldsymbol{P}_{\boldsymbol{liq}}\boldsymbol{+0.5*}\boldsymbol{P}_{\boldsymbol{ice}}\boldsymbol{)}$ (S17) | Q_P_ [MJ m^-2^ d^-1^]: Head advected to the snowpack from rain [*Wigmosta et al.,* 1994]; ρ_w_ [1000 kg m^-3^]: density of water; C_w_ [4.2 E-3 MJ kg^-1^ ⁰C^-1^]: specific heat capacity of water. |
| $\boldsymbol{Q}_{\boldsymbol{S}}\boldsymbol{=}\frac{\boldsymbol{c}_{\boldsymbol{air}}\boldsymbol{*(}\boldsymbol{T}_{\boldsymbol{a}}\boldsymbol{-}\boldsymbol{T}_{\boldsymbol{sn}}\boldsymbol{)}}{\boldsymbol{r}_{\boldsymbol{a}}}$ (S18) | Q_S_ [MJ m^-2^ d^-1^]: Sensible heat exchange with the atmosphere and the snowpack [*Walter et al.,* 2005]; Where c_air_ [1.29 E-3 MJ m^-3^ ⁰C^-1^]: heat capacity of air; r_a_ [d m^-1^]: resistance to heat transfer. |
| $\boldsymbol{r}_{\boldsymbol{a}}\boldsymbol{=}\frac{\boldsymbol{ln}\left( \frac{\boldsymbol{(}\boldsymbol{z}_{\boldsymbol{u}}\boldsymbol{-}\boldsymbol{d}_{\boldsymbol{s}}\boldsymbol{+}\boldsymbol{z}_{\boldsymbol{ms}}\boldsymbol{)}}{\boldsymbol{z}_{\boldsymbol{ms}}} \right)\boldsymbol{ln}\left( \frac{\mathbf{}\boldsymbol{(}\boldsymbol{z}_{\boldsymbol{u}}\boldsymbol{-}\boldsymbol{d}_{\boldsymbol{s}}\boldsymbol{+}\boldsymbol{z}_{\boldsymbol{hs}}\boldsymbol{)}}{\boldsymbol{z}_{\boldsymbol{hs}}} \right)}{\boldsymbol{k}^{\boldsymbol{2}}\boldsymbol{W}_{\boldsymbol{can}}}\frac{\boldsymbol{1}}{\boldsymbol{86400}}$ (S19) | r_a_ in [*Walter et al.,* 2005]; z_u_ [m]: measurement height of the climate variables; d_s_ [0 m]: zero-plane displacement for snow; z_ms_ [0.001 m]: momentum roughness for snow; z_hs_ [0.0002 m]: heat and vapour roughness parameter for snow; k [0.41]: von Karman’s constant. |
| $\boldsymbol{Q}_{\boldsymbol{L}}\boldsymbol{=}\boldsymbol{\lambda}_{\boldsymbol{v}}\frac{\boldsymbol{\rho}_{\boldsymbol{a}}\boldsymbol{-}\boldsymbol{\rho}_{\boldsymbol{sa}}}{\boldsymbol{r}_{\boldsymbol{a}}}$ (S20) | Q_L_ [MJ m^-2^ d^-1^]: latent heat flux [*Walter et al.,* 2005]; λ_v_ [2.8 MJ kg^-1^]: latent heat of vaporization; ρ_a_ and ρ_sa_ [kg m^-3^]: vapor density of the air and snow surface, respectively. |
| $\boldsymbol{\rho}_{\boldsymbol{a}}\boldsymbol{=}\frac{\boldsymbol{p}_{\boldsymbol{sat}}}{\left( \boldsymbol{T}_{\boldsymbol{a}}\boldsymbol{+273.15} \right)\boldsymbol{*}\boldsymbol{R}_{\boldsymbol{v}}}$ (S21)  $\boldsymbol{\rho}_{\boldsymbol{sat}}\boldsymbol{=}\frac{\mathbf{exp}\boldsymbol{(16.78*}\boldsymbol{T}_{\boldsymbol{sn}}\boldsymbol{-116.8)}}{{\boldsymbol{(T}_{\boldsymbol{sn}}\boldsymbol{+273.3)}}^{\boldsymbol{2}}\boldsymbol{*}\boldsymbol{R}_{\boldsymbol{v}}}$ (S22) | ρ_a_ from [*Walter et al.,* 2005]; R_v_ [4.63 E-3 MJ kg^-1^ ⁰C^-1^]: thermodynamic constant for water vapor and p_sat_ [-]: vapor pressure. |
| $\boldsymbol{p}_{\boldsymbol{sat}}\boldsymbol{=0.6108*}\exp\left( \frac{\boldsymbol{17.27*}\boldsymbol{T}_{\boldsymbol{a}}}{\boldsymbol{237.7+}\boldsymbol{T}_{\boldsymbol{a}}} \right)\boldsymbol{*10*}\frac{\boldsymbol{RH}}{\boldsymbol{100\%}}$ (S23) | RH [%]: relative humidity of the air. |
| $\boldsymbol{E}_{\boldsymbol{sum}}\boldsymbol{=}\boldsymbol{R}_{\boldsymbol{sn}}\boldsymbol{+}\boldsymbol{L}_{\boldsymbol{net}}\boldsymbol{+}\boldsymbol{Q}_{\boldsymbol{P}}\boldsymbol{+}\boldsymbol{Q}_{\boldsymbol{S}}\boldsymbol{+}\boldsymbol{Q}_{\boldsymbol{L}}$ (S24) | E_sum_ [MJ m^-2^ d^-1^]: sum of energy inputs into the snowpack. Positive sum means snow pack is receiving energy. If E_sum_>0 and temperature T_sn_ < 0, energy is diverted to heat the snowpack. |
| $\boldsymbol{Q}_{\boldsymbol{M}}\boldsymbol{=}\begin{matrix} \boldsymbol{E}_{\boldsymbol{sum}}\boldsymbol{-}\frac{\boldsymbol{0-}\boldsymbol{T}_{\boldsymbol{sn}}}{\boldsymbol{C}_{\boldsymbol{i}}\boldsymbol{*SWE*}\boldsymbol{\rho}_{\boldsymbol{w}}} & \boldsymbol{if}\boldsymbol{E}_{\boldsymbol{sum}}\boldsymbol{>0 \&}\boldsymbol{T}_{\boldsymbol{sn}}\boldsymbol{<0} \\ \boldsymbol{E}_{\boldsymbol{sum}} & \boldsymbol{if}\boldsymbol{E}_{\boldsymbol{sum}}\boldsymbol{>0 \&}\boldsymbol{T}_{\boldsymbol{sn}}\boldsymbol{=0} \\ \boldsymbol{-\rho}_{\boldsymbol{w}}\boldsymbol{*}\boldsymbol{\lambda}_{\boldsymbol{f}}\boldsymbol{*}\boldsymbol{W}_{\boldsymbol{liq}} & \boldsymbol{if}\boldsymbol{E}_{\boldsymbol{sum}}\boldsymbol{<0} \end{matrix}$  (S25) | Q_M_ [MJ m^-2^ d^-1^]: energy available for melting snow or refreezing liquid water in snowpack; C_i_ [MJ kg^-1^ ⁰C^-1^]: specific heat capacity for ice; W_liq_ [m]: liquid water retained by the snowpack. Amount of energy diverted to melting and refreezing are limited by the available SWE and W_liq_, respectively. |
| $\boldsymbol{T}_{\boldsymbol{sn}}\boldsymbol{=}\boldsymbol{T}_{\boldsymbol{sn}}\boldsymbol{+}\frac{\boldsymbol{E}_{\boldsymbol{sum}}\boldsymbol{-}\boldsymbol{Q}_{\boldsymbol{M}}}{\boldsymbol{C}_{\boldsymbol{i}}\boldsymbol{*SWE*}\boldsymbol{\rho}_{\boldsymbol{w}}}$ (S26) | To aid numerical stability of the solution we assume that T_sn_ always ≤0; for low snow packs (SWE <0.05 m) T_sn_=T_a_; and is situations where lots of energy is being lost from the snowpack, snow temperature decrease is limited by T_sn_ < -4, T_sn_ = max(T_sn_, T_a_). |
| $\boldsymbol{SWE}_{\boldsymbol{j}}\boldsymbol{=}{\boldsymbol{W}_{\boldsymbol{ice}}}_{\boldsymbol{j-1}}\boldsymbol{+}{\boldsymbol{W}_{\boldsymbol{liq}}}_{\boldsymbol{j-1}}{\boldsymbol{+P}_{\boldsymbol{thru}}}_{\boldsymbol{j}}\boldsymbol{+}{\boldsymbol{P}_{\boldsymbol{unl}}}_{\boldsymbol{j}}\boldsymbol{+}{\boldsymbol{P}_{\boldsymbol{liq}}}_{\boldsymbol{j}}\boldsymbol{-}{\boldsymbol{S}_{\boldsymbol{melt}}}_{\boldsymbol{j}}\boldsymbol{+}{\boldsymbol{E}_{\boldsymbol{snow}}}_{\boldsymbol{j}}$  (S27) | SWE for time step j; W_ice_ [m]: ice in the snowpack; W_liq_ [m]: liquid water retained in the snowpack; P_thru_ [m]: throughfall of snow precipitation; P_unl_ [m]: unloading of snow from interception storage; S_melt_ [m]: amount of snowmelt or refreezing of liquid water; E_snow_ [m]: snow sublimation/ condensation of water vapor. |
| $\boldsymbol{S}_{\boldsymbol{melt}}\boldsymbol{=}\frac{\boldsymbol{Q}_{\boldsymbol{M}}}{\boldsymbol{\lambda}_{\boldsymbol{f}}\boldsymbol{*}\boldsymbol{\rho}_{\boldsymbol{w}}}$ (S28) | S_melt_ [m]: Snow melt or refreezing |
| $\boldsymbol{E}_{\boldsymbol{snow}}\boldsymbol{=}\frac{\boldsymbol{Q}_{\boldsymbol{L}}}{\boldsymbol{\lambda}_{\boldsymbol{v}}\boldsymbol{*}\boldsymbol{\rho}_{\boldsymbol{w}}}$ (S29) | Sublimation/condensation E_snow_ [m] is approximated as the latent heat exchange |
| $\boldsymbol{W}_{\boldsymbol{out}}\boldsymbol{=}\boldsymbol{W}_{\boldsymbol{liq}}\boldsymbol{+}\boldsymbol{P}_{\boldsymbol{liq}}\boldsymbol{+}\boldsymbol{S}_{\boldsymbol{melt}}\boldsymbol{-r*}\boldsymbol{W}_{\boldsymbol{ice}}$ (S30) | W_out_ [m]: Snowmelt runoff; r [-]: water retention capacity of the snowpack. |
| $\boldsymbol{I}_{\boldsymbol{sn}}\boldsymbol{=}\boldsymbol{I}_{\boldsymbol{sn(i-1)}}\boldsymbol{+0.7*}\left( \boldsymbol{I}_{\boldsymbol{max}}\boldsymbol{-}\boldsymbol{I}_{\boldsymbol{sn(i-1)}} \right)\boldsymbol{*}\left( \boldsymbol{1-}\boldsymbol{e}^{\boldsymbol{-}\frac{\boldsymbol{P}_{\boldsymbol{ice}}}{\boldsymbol{I}_{\boldsymbol{max}}}} \right)$  (S31) | I_sn_ [kg m^-2^]: canopy snow interception [*Hedstrom and Pomeroy,* 1998; *Liston and Elder,* 2006]; I_max_ [kg m^-2^]: maximum interception storage; P_ice_ [m] is converted to units [kg m^-2^] by constant snow density ρ_sn_ of 150 [kg m^-3^]; |
| $\boldsymbol{I}_{\boldsymbol{max}}\boldsymbol{=4.4*LAI}$ (S32) | I_max_ in [*Hedstrom and Pomeroy,* 1998] |
| $\boldsymbol{S}_{\boldsymbol{thru}}\boldsymbol{=}\boldsymbol{P}_{\boldsymbol{ice}}\boldsymbol{-(}\boldsymbol{I}_{\boldsymbol{sn}}\boldsymbol{-}\boldsymbol{I}_{\boldsymbol{sn}\left( \boldsymbol{i-1} \right)}\boldsymbol{)}$ (S33) | S_thru_ [kg m^-3^]: Throughfall of snow precipitation |
| $\boldsymbol{S}_{\boldsymbol{unl}}\boldsymbol{=}\boldsymbol{U}_{\boldsymbol{dd}}\boldsymbol{*}\boldsymbol{T}_{\boldsymbol{a}}$ (S34) | S_unl_ [kg m^-2^]: unloading of snow from interception storage [*Liston and Elder,* 2006]; U_dd_ [5 kg d^-1^ m^-2^ ⁰C^-1^]: unloading factor. |

Table S1. Model equations for simulating spatially distributed snow interception, accumulation, and melt.

| day | Site | avg d18O [‰] | sd d18O [‰] |
| --- | --- | --- | --- |
| 10/04/2004 | Krycklan | -17.9 | 2.3 |
| 13/04/2004 | Krycklan | -18 | 0.6 |
| 15/04/2004 | Krycklan | -16.1 | 1.5 |
| 17/04/2004 | Krycklan | -15.4 | 1.2 |
| 18/04/2004 | Krycklan | -15.6 | 1.4 |
| 19/04/2004 | Krycklan | -15.6 | 3.2 |
| 20/04/2004 | Krycklan | -15.7 | 2.7 |
| 21/04/2004 | Krycklan | -15.5 | 1.1 |
| 23/04/2004 | Krycklan | -15.8 | 2.9 |
| 26/04/2004 | Krycklan | -15.4 | 3.8 |
| 27/04/2004 | Krycklan | -15.9 | 1.5 |
| 29/04/2004 | Krycklan | -15.6 | 3.1 |
| 03/05/2004 | Krycklan | -15.1 | 1.5 |
| 08/05/2004 | Krycklan | -12.8 | 3.4 |
| 19/05/2004 | Krycklan | -10.9 | 2.1 |
| 30/05/2004 | Krycklan | -10.5 | 2.4 |
| 13/04/2010 | Krycklan | -19.3 | 4.6 |
| 15/04/2010 | Krycklan | -19.5 | 2.4 |
| 19/04/2010 | Krycklan | -19.7 | 0.1 |
| 22/04/2010 | Krycklan | -19.4 | 2.8 |
| 26/04/2010 | Krycklan | -18.9 | 3 |
| 29/04/2010 | Krycklan | -18.1 | 2 |
| 03/05/2010 | Krycklan | -16.2 | 2 |
| 06/05/2010 | Krycklan | -17.1 | 2.7 |
| 10/05/2010 | Krycklan | -16.7 | 5.5 |
| 12/05/2010 | Krycklan | -16.7 | 0.1 |
| 14/05/2010 | Krycklan | -13.6 | 7.2 |
| 17/05/2010 | Krycklan | -14.4 | 8.8 |
| 20/04/2012 | Krycklan | -17.1 | 2.7 |
| 26/04/2012 | Krycklan | -16.6 | 2 |
| 30/04/2012 | Krycklan | -16 | 5.5 |
| 02/05/2012 | Krycklan | -15.4 | 0.8 |
| 04/05/2012 | Krycklan | -14.9 | 0.9 |
| 07/05/2012 | Krycklan | -14.7 | 2.7 |
| 09/05/2012 | Krycklan | -14.4 | 3.1 |
| 11/05/2012 | Krycklan | -14.9 | 2.7 |
| 14/05/2012 | Krycklan | -14.7 | 2 |
| 16/05/2012 | Krycklan | -13.6 | 1.6 |
| 18/05/2012 | Krycklan | -13.2 | 2.6 |
| 21/05/2012 | Krycklan | -12 | 10.4 |
| 23/05/2012 | Krycklan | -13.7 | 6.3 |
| 11/01/2003 | Bogus | -16.8 | 2.3 |
| 17/01/2003 | Bogus | -16.2 | 0.8 |
| 01/02/2003 | Bogus | -15 | 0.7 |
| 05/02/2003 | Bogus | -16.3 | 0.7 |
| 27/02/2003 | Bogus | -17.9 | 1.1 |
| 12/03/2003 | Bogus | -16.1 | 1.1 |
| 13/03/2003 | Bogus | -16.5 | 0.7 |
| 15/03/2003 | Bogus | -15.7 | 0.7 |
| 21/03/2003 | Bogus | -12.6 | 0.7 |
| 30/03/2003 | Bogus | -16.7 | 0.9 |
| 08/04/2003 | Bogus | -16 | 1.2 |
| 14/04/2003 | Bogus | -13.9 | 0.6 |

Table S2. The average (avg) and standard deviation (sd) in δ^18^O for snowmelt lysimeter samples at Bogus and Krycklan sites.
